# Molecular Characterization and Expression Analysis of Chloroplast Protein Import Components in Tomato (*Solanum lycopersicum*)

**DOI:** 10.1371/journal.pone.0095088

**Published:** 2014-04-21

**Authors:** Jianmin Yan, James H. Campbell, Bernard R. Glick, Matthew D. Smith, Yan Liang

**Affiliations:** 1 College of Horticulture, Northwest A&F University, Yangling, Shaanxi, P.R. China; 2 Department of Biology, University of Waterloo, Waterloo, Ontario, Canada; 3 Department of Biology, Wilfrid Laurier University, Waterloo, Ontario, Canada; University of California - Davis, United States of America

## Abstract

The translocon at the outer envelope membrane of chloroplasts (Toc) mediates the recognition and initial import into the organelle of thousands of nucleus-encoded proteins. These proteins are translated in the cytosol as precursor proteins with cleavable amino-terminal targeting sequences called transit peptides. The majority of the known Toc components that mediate chloroplast protein import were originally identified in pea, and more recently have been studied most extensively in Arabidopsis. With the completion of the tomato genome sequencing project, it is now possible to identify putative homologues of the chloroplast import components in tomato. In the work reported here, the Toc GTPase cDNAs from tomato were identified, cloned and analyzed. The analysis revealed that there are four Toc159 homologues (slToc159-1, -2, -3 and -4) and two Toc34 homologues (slToc34-1 and -2) in tomato, and it was shown that tomato Toc159 and Toc34 homologues share high sequence similarity with the comparable import apparatus components from Arabidopsis and pea. Thus, tomato is a valid model for further study of this system. The expression level of Toc complex components was also investigated in different tissues during tomato development. The two tomato Toc34 homologues are expressed at higher levels in non-photosynthetic tissues, whereas, the expression of two tomato Toc159 homologues, slToc159-1 and slToc159-4, were higher in photosynthetic tissues, and the expression patterns of slToc159-2 was not significantly different in photosynthetic and non-photosynthetic tissues, and slToc159-3 expression was limited to a few select tissues.

## Introduction

Plastids, whose evolutionary history can be traced to free-living cyanobacteria that were incorporated into a host cell through endosymbiosis, are organelles that perform essential metabolic and signaling processes in all plant cells [1], [2]. There are many different types of plastids, all of which develop from proplastids and are interconvertible depending on the tissue in which they reside and the environmental conditions. For example, proplastids can develop into chromoplasts, which are, in turn, able to re-differentiate into green chloroplasts under certain conditions [1], [2]. The biogenesis and maintenance of specific plastid types in different tissues relies on the coordinated expression and import of thousands of nucleus-encoded proteins [3]–[5]; the vast majority of plastid proteins are encoded in the nucleus, as most of the genes from the original endosymbiont have been transferred to the nuclear genome during the evolutionary transition from free-living cyanobacteria to semi-autonomous organelle [6]. It has been estimated that approximately 95% of plastid proteins, representing ∼1900–2500 distinct proteins [7], are encoded in the nucleus, synthesized as precursor proteins (preproteins) in the cytosol and imported post-translationally into the organelle [8], [9]. It is well established that import is facilitated by interactions between the intrinsic N-terminal transit peptide of the nuclear-encoded preproteins and a common recognition and translocation machinery located in the chloroplast double-membrane envelope [10].

The chloroplast protein import apparatus consists of translocons at the outer and inner envelope membranes of the chloroplast, called the Toc and Tic complexes, respectively. The core components of the Toc complex are generally agreed to be Toc159, Toc34 and Toc75 [6], which were first identified and characterized in pea [9]. Toc159 and Toc34 are related GTPases (the Toc GTPases) that possess highly similar sequences within their GTP-binding domains, and are believed to act as receptor components responsible for the recognition of preproteins and the initiation of import [11]–[14]. Toc159 has a tripartite domain structure, consisting of a 52-kD, C-terminal membrane-anchor domain (M-domain), a central GTP binding domain (G-domain), and an intrinsically disordered N-terminal acidic domain (A-domain) [12], [15], [16]. In *Arabidopsis*, four Toc159 homologues have been identified: atToc159, atToc132, atToc120 and atToc90 [12], [17].

Toc34, like Toc159, is a GTP-binding component of the import machinery. It is anchored in the outer membrane by a transmembrane α-helix near the C-terminus [18], which characterizes it as being a tail-anchored protein [19]. The GTPase domain, which comprises the majority of the protein, is exposed to the cytosol [20]–[22], and contains four conserved GTP-binding domains (G1-G4) [18], [20], [23]. In *A. thaliana*, two homologues of Toc34 are present, named atToc34 and atToc33 [22], [24]–[26].

Toc75 is a β-barrel that serves as the protein-conducting channel through the outer envelope [11], [22], [27] and comprises a key part of the Toc core complex together with Toc159 and Toc34. The Toc/Tic complexes are best characterized in pea (*Pisum sativum*) and *Arabidopsis thaliana* [18], [20], [28]–[30]. The recent completion of the tomato genome sequencing project [31], provides the opportunity to extend the study of this system to tomato. The expression profile and import of nucleus-encoded plastid proteins is affected by both the type and developmental stage of the particular plastid. While the different types of plastids are inter-convertible [32], each type performs specific functions [4]. For example, chloroplasts are found in green tissues and are the site of photosynthesis; chromoplasts are found in non-green, non-photosynthetic tissues such as fruits, roots, and flower petals; and amyloplasts are non-photosynthetic plastids located in roots [4]. While these non-green plastids don’t perform photosynthesis, they do have important functions in lipid and starch storage, and are the site of other important biochemical processes such as amino acid and lipid biosynthesis [4]. Most biochemical analyses of the plastid protein import machinery have used chloroplasts isolated from Arabidopsis and pea [13], [14], [22], [28], [33], [34]; other types of plastids have not been studied to nearly the same extent. One of the few studies on protein import into non-photosynthetic plastids used chromoplasts isolated from red bell peppers [35]. An advantage offered by a model system such as tomato, whose genome has now been sequenced, compared to Arabidopsis, is the abundance of non-green tissues such as ripe fruit, where non-photosynthetic plastids (i.e. chromoplasts) are abundant. The availability of different plastid types should allow for a more comprehensive comparison of the composition of the Toc and Tic machineries, how their activities differ among plastid types, and how the machinery is involved in the differentiation or biogenesis of functionally different plastid types. Here, the genes for the Toc GTPase homologues in tomato have been identified, the expression of these genes during tomato development has been analyzed using quantitative Real-Time PCR which facilitates comparison of the gene expression of components of the plastid protein import apparatus in various plant tissues during development.

## Materials and Methods

### Identification and Isolation of TocGTPase Genes from Tomato

The Arabidopsis protein and cDNA sequences from NCBI (National Centre for Biotechnology Information) were used as query sequences to search for putative Toc GTPase nucleotide and protein sequences from tomato, using the SOL Genomics Network (SGN; http://www.sgn.cornell.edu). NCBI ORF finder was used to find putative open reading frames (http://www.ncbi.nlm.nih.gov/gorf/gorf.html) and functional domains were determined using BLASTp available through NCBI. Molecular weights and pI’s were predicted using Protparam (http://web.expasy.org/protparam/). The coding region of slToc34-1, slToc34-2, slToc159-1, slToc159-2, slToc159-3 and slToc159-4 were amplified separately by PCR using primers designed based on the 5′ and 3′ ends of their predicted sequences (Table 1).

**Table 1:** Details of Primers and Amplicons for Each of the Evaluated Genes.

Gene name	Gene code*	Primer(5′ to 3′)	Amplicon(bp)	Tm	GC%
Actin	TC194780	GAAATAGCATAAGATGGCAGACG	159	63.5	43.5
		ATACCCACCATCACACCAGTAT		61.7	45.5
CAC	SGN-U314153	CCTCCGTTGTGATGTAACTGG	173	64.2	52.4
		ATTGGTGGAAAGTAACATCATCG		63.6	39.1
slToc34-1	Solyc03g095220.2.1	CCCTCCTGATGGATTGACTT	307	62.8	50.0
		CACTTTGCCCCTATTGTTTG		61.8	45.5
slToc34-2	Solyc05g052160.2.1	TTGGATAGGCAGATTGTAAAGG	99	61.7	40.9
		AGGAGGGGAGACCTGAGCAT		66.3	50.0
slToc159-1	Solyc09074940.1.1	GCCATTGCCAGTCGTTTC	231	64.3	55.6
		TGTAACAGAAATTCCGCAAG		60.3	40.0
slToc159-2	Solyc01g080780.2.1	TGATTACGATGACCTTCCACC	136	63.6	47.6
		CTCCCTCCATTGTTTCTTCTG		62.5	47.6
slToc159-3	Solyc11g043010.1.1	CTGATAACCCAACTCATAGATACCG	346	63.3	44.0
		ACTTTCACCCCACTTGTCATAAC		62.8	43.5
slToc159-4	XM_004230964	CTTCGCAGTGAGACCAGA	239	56.2	55.6
		AGGAAACGACCAAGAGGA		57.0	50.0
pE1a	Solyc12g009410.1.1	ACAACCTGGCAAGTGAAGC	106	62.9	52.6
		CTACGAACAGGATGAGGGC		62.2	57.9
RbcS	Solyc03g034220.2.1	GTTGCCTATGTTTGGGTGC	79	62.9	52.6
		TGCTTGTGGGTATGCCTTT		63.0	47.4
slToc34-1C	Solyc03g095220.2.1	GGGTTTCA TATG GGGAAAGGCGGTGTTG	780	62.8	62.5
		GCTTACGAGCT CTTATGCCCATGAAGGCCTG		65.8	52.6
slToc34-2C	Solyc05g052160.2.1	GGAATCCA TATGGCATCTCAGGTGATAAGAG	906	54.6	47.4
		CGTTACGTCGACTTATGCCCATGAAGGTCTGTTC		52.4	45.5
slToc159-2C	Solyc01g080780.2.1	CGCACGC CATGGAACTTATCATAGATCAGTCG	3489	59.6	35.0
		CACGGCGAGCTCCTATATTAAGTTCTTTCCACTTGC		56.0	32.0

*Gene code from GenBank database or Sol Genomics Network (SGN).

The sizes of the PCR products were verified using 1% agarose gels. The amplified fragments of the tomato Toc34 homologues (slToc34-1, slToc34-2) and slToc159-2 were cloned into the pGEM-T easy (Promega) and pBluescript vectors, respectively. Sequencing was done by the Advanced Analysis Centre Genomics Facility, University of Guelph (Guelph, ON, Canada).

### The Analysis of Chromosomal Localization and Gene Structure

The chromosomal localization data and gene structures of the tomato Toc GTPases were analyzed by comparing the full-length cDNA sequences against the *Solanum lycopersicum* genome sequences (http://mips.helmholtz-muenchen.de/plant/tomato/searchjsp/index.jsp). The exon–intron organizations were mapped using Gene Structure Display Server (GSDS, http://gsds.cbi.pku.edu.cn/) [36].

### Sequence Alignment and Phylogenetics

Similarity sequence analysis at the DNA and protein levels were performed using the blastn and blastp programs, respectively, from the National Center for Biotechnology Information (http://www.ncbi.nlm.nih.gov/blast). Multiple sequence alignments were built, and phylogenetic trees were determined, using the neighbor-joining algorithm, followed by 1000 bootstrap replicates, using DNAMAN software (Version 5.2.2.0, Lynnon Biosoft, USA).

### Plant Material

Tomato plants (*Solanum lycopersicum* cv. Heinz-722) were grown in Sunshine Mix #4 (Sun Gro Horticulture Canada Ltd.) in 9 cm diameter plastic pots in a greenhouse from May to September at Wilfrid Laurier University (Waterloo, ON), and were watered every day as needed.

### Tissue Collection

Tissue samples were collected from tomato plants at different developmental stages as follows. Cotyledons (C) were collected 20 days after the seeds were sowed. young leaf (YL) samples were harvested at the 5-leaf stage. Floral buds (FB) (about 8 mm) were collected one day before flower opening. Flowers were collected the first day after flower opening (OF). Green fruit (GF) tissues were harvested after 15 days of fruit setting. Red fruit (RF) samples were harvested at the totally ripe red fruit stage (seeds were removed). Roots (R) were excised at the 5-leaf stage. After collection, samples were immediately frozen in liquid N_2_ and stored at −80°C until they were utilized for RNA isolation.

### RNA Isolation and Reverse Transcription

Total RNA was isolated from tissue samples using Trizol, following the manufacturer’s recommendations (Invitrogen). Residual DNA was removed by digestion with RNase-free DNase (Invitrogen). The concentration of total RNA was measured with a Nano Drop spectrophotometer (Thermo Scientific, USA). RNA was quantified by measuring the absorbance at 260 nm, its purify was assessed based on the A_260_/A_280_ ratio, and RNA integrity was examined using 1% agarose gel electrophoresis.

The cDNA samples for quantitative real-time PCR (qRT-PCR) experiments were synthesized using an oligo(dT)_20_ primer and 1 µg of total RNA with the superscript first-strand synthesis system for RT-PCR (Invitrogen). A mixture of the cDNA samples from cotyledons, young leaves, flower buds, open flowers, green fruits, red fruits and roots were diluted using a 10-fold dilution series until 1∶10^5^. The serially diluted cDNA was used for making a standard curve and selecting the optimal concentration of each cDNA to use for the gene expression study.

### Quantitative PCR Primer Design

Primer pairs used for qRT-PCR were designed using Primer5 software (PREMIER Biosoft International, Silicon Valley, USA). In a series of initial experiments, the performance of the designed primers (Table1) was tested by real-time PCR using a mixture of cDNA templates from all tissues.

### Quantitative Real-Time PCR Reaction

Quantitative real-time PCR was performed using SsoFast EvaGreen Supermix (Bio-Rad) and run in triplicate in 48-well plates with an MJ Mini Thermal Cycler (Bio-Rad). The cycling profile consisted of 95°C for 1 min, followed by 40 cycles of 5 s at 95°C and 20 s at 60°C, as recommended by the manufacturer. The amplification process was immediately followed by a melting curve analysis steps of 0.5°C every 10 s from 65°C to 95°C. Baseline and threshold cycles (Ct) were automatically determined using the Bio-Rad CFX manager software (Bio-Rad). Here, we selected a classical housekeeping gene, Actin, and a novel housekeeping gene, CAC [37] (Table1) as internal controls for constitutive expression in various tissues.

## Results

### Identification and Cloning of Tomato Toc GTPase cDNAs

To identify putative Toc GTPase sequences from tomato, a BLASTn search was conducted against the *Solanum lycopersicum* genome sequences (http://mips.helmholtz-muenchen.de/plant/tomato/searchjsp/index.jsp) using the amino acid sequences of the Arabidopsis Toc GTPases as query sequences in 2010. The five sequences that were obtained from the tomato genome database with high homology to the Arabidopsis sequences were designated slToc34-1, slToc34-2, slToc159-1, slToc159-2 and slToc159-3. Based on the sequences from the tomato genome sequencing project, the coding regions of all five genes were amplified by PCR. Using this approach, three of the five cDNAs, slToc34-1, slToc34-2 and slToc159-2, were successfully cloned. These cDNAs have also been reported recently following an automated gene prediction exercise using the NCBI: Gnomon method, which is supported by mRNA and EST evidence [36]. This analysis also predicted five Toc GTPases from *Solanum lycopersicum*, which are annotated as being chloroplast-translocase 34 or 159 on the NCBI website; specific or definitive names were not provided [36]. Here, we tentatively designate the five NCBI-identified tomato Toc GTPases as slToc34-like-1 (XM_004235160), slToc34-like-2 (XM_004239929), slToc159-like-1 (XM_004247489), slToc159-like-2 (XM_004229523) and slToc159-like-3 (XM_004230964).

**Figure 1:**
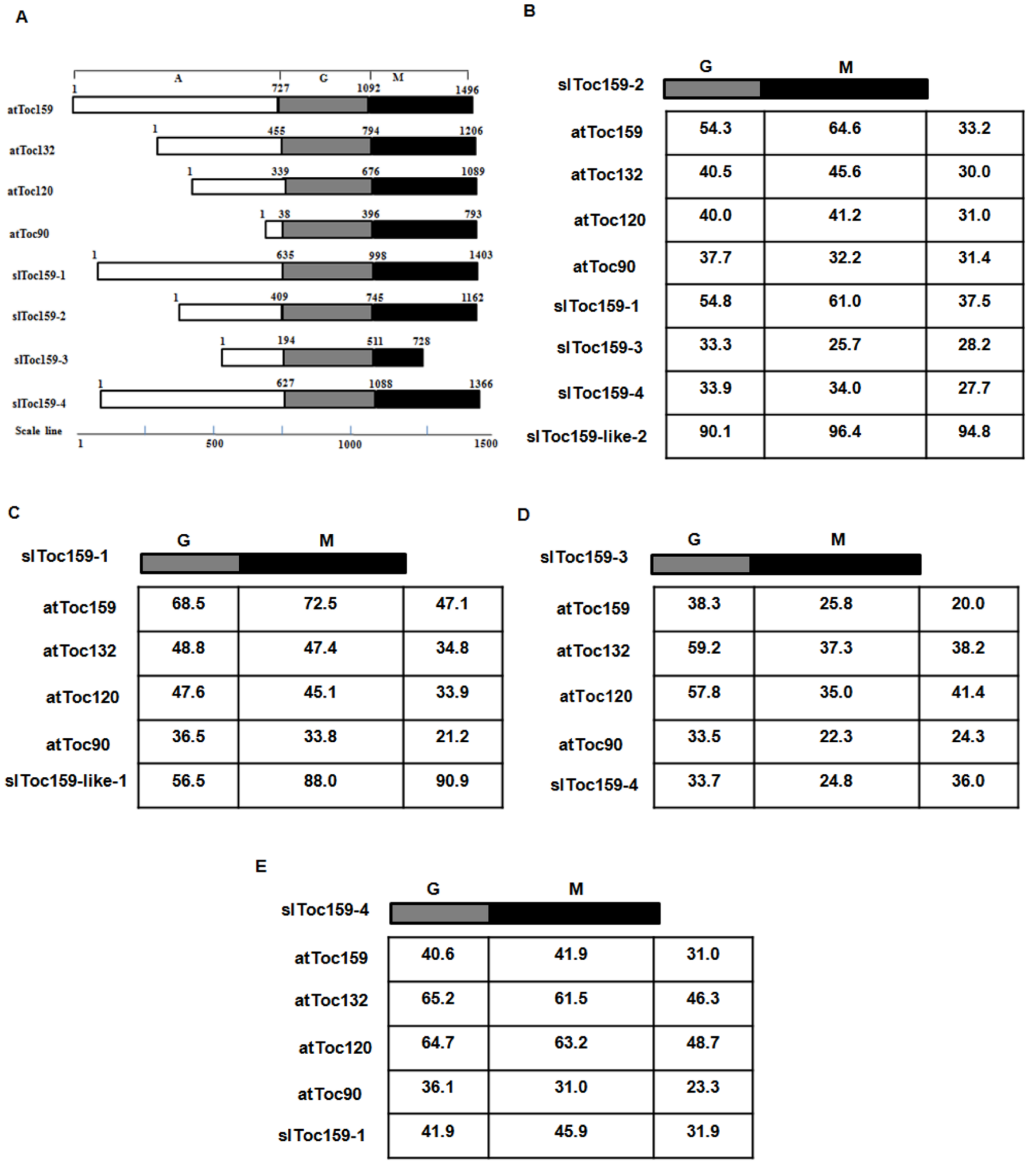
Structural comparison of the members of the Toc159 import receptor family. (A) Alignment of linear representations of the Toc159 family. The positions of the acidic domains (A, white boxes), the GTPase domains (G, gray boxes), and the membrane anchor domains (G, black boxes) are shown. The amino acid numbers above each protein indicate the borders of each domain. (B) Comparison of the amino acid sequence identity between the domains of each protein (G+M domain) relative to slToc159-2 (G+M domain). (C) Comparison of the amino acid sequence identity between the domains of each protein (G+M domain) relative to slToc159-1 (G+M domain). (D) Comparison of the amino acid sequence identity between the domains of each protein (G+M domain) relative to slToc159-3 (G+M domain). (E) Comparison of the amino acid sequence identity between the domains of each protein (G+M domain) relative to slToc159-4 (G+M domain). The last column of each table reports the overall identity of the each full-length protein, including the A-domain. The tables report the percentage of identity.

**Figure 2:**
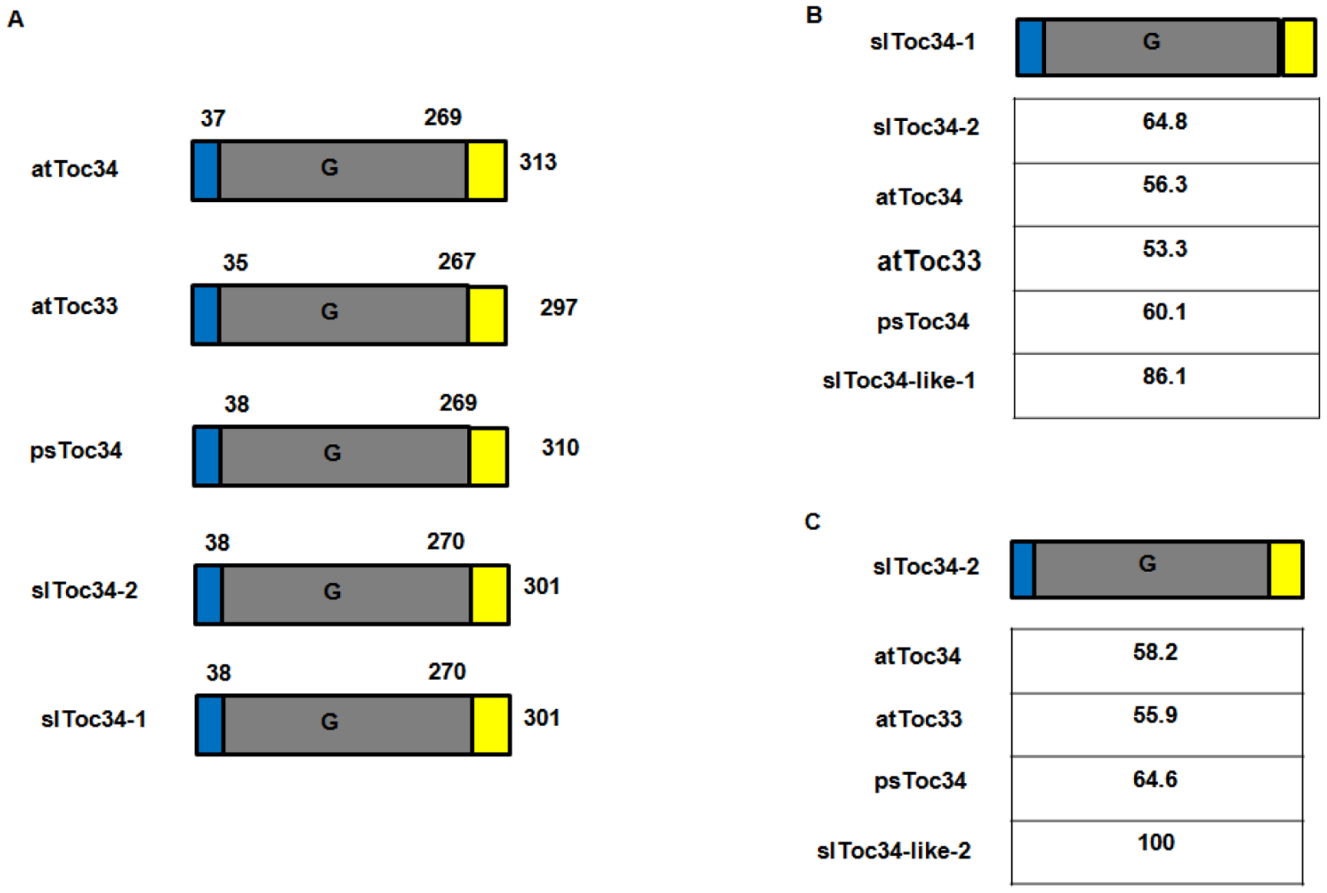
Structural comparison of the members of the Toc34 import receptor family. (A) Alignment of linear representations of the Toc34 family. G, GTP-binding domain (shaded in gray); regions other than the G domains are given in blue and yellow, respectively; The amino acid numbers above each protein indicate the borders of each domain. (B) Comparison of the amino acid sequence identity between the domains of each protein relative to slToc34-1. (C) Comparison of the amino acid sequence identity between the domains of each protein relative to slToc34-2. The table presents the percentage of identity.

We compared the deduced amino acid sequences of the tomato Toc GTPases that were identified here with those identified in the NCBI analysis (Fig. 1B and E; Fig. 2B and C; Fig. S1, S2, S3, S4, and S5) using DNAMAN software (Version 5.2.2.0, Lynnon Biosoft, USA). This comparison revealed that slToc34-2 and slToc34-like-2 are identical (Fig. 2C; Fig. S2). On the other hand, differences were observed in the sequences of slToc34-1, slToc159-1 and slToc159-2 identified in the present analysis as compared to the corresponding sequences from NCBI (i.e. slToc34-like-1, slToc159-like-1 and slToc159-like-2, respectively). slToc34-1 is shorter than slToc34-like-1 by 42 amino acids at the N-terminus (Fig. S1); they share 86.1% overall sequence identity (Fig. 2B). slToc159-1 is longer than slToc159-like-1 by 128 amino acids in the A-domain (Fig. S3); they share 90.9% overall amino acid identity (Fig. 1C). slToc159-2 is shorter than slToc159-like-2 by 60 amino acids at the N-terminus (Fig. S4); they share 94.8% overall amino acid identity (Fig. 1B). Each of the closely-related Toc GTPases identified in the present analysis, those revealed by the NCBI gene prediction analysis, is located on the same chromosome. Specifically, slToc34-1 (and slToc34-like-1) is on chromosome 3; slToc34-2 (and slToc34-like-2) is on chromosome 5; slToc159-1 (and slToc159-like-1) is on chromosome 9; slToc159-2 (and slToc159-like-2) is on chromosome 1. It is therefore concluded that each comparable Toc GTPase in this group that was identified in both the present analysis and the NCBI analysis is the same gene, and they will be referred to as slToc34-1, slToc34-2, slToc159-1 and slToc159-2.

slToc159-3 (Solyc11g043010.1.1) and slToc159-like-3 (XM_004230964) share only share 36.0% overall identity (Fig. 1D; Fig. S5), and are located on chromosome numbers 11 and 1, respectively. Thus, while they are both likely to be Toc159 homologues, corresponding to two different tomato Toc159 GTPases, the genome analysis done here as well as the NCBI gene prediction analysis each missed one potential Toc159-related GTPase, so that there are potentially four Toc159 homologues in tomato. These two are henceforth considered to be separate Toc159 homologues. We have designated the homologue identified in the present analysis slToc159-3 and the homologue from the NCBI analysis (originally called slToc159-like-3) as slToc159-4.

A summary of the tomato Toc GTPase cDNAs and the predicted proteins that they encode is shown Table 2. The cDNA and amino acid sequences of each identified tomato Toc GTPase are provided in Data S1, S2, S3, and S4.

**Table 2:** Gene analysis of Toc GTPase in tomato (*Solanum lycopersicum*).

Gene name	Accession number	Chromosome a	5′UTR b	Exton c	3′UTR d	ORF length(bp) (aa) e	pI f	MW (kDa)^g^
slToc34-1	XM_004235160	3	1–470	471–569,1460–1567,1662–1786,2314–2578,3820–3888,3986–4147,4232–4309	4310–4609	906 (301)	9.54	33.32
slToc34-2	Solyc05g052160.2.1	5	–	1–99, 291–398,490–612,756–1022,2065–2133,2213–2374,2482–2559	2560–2772	906 (301)	9.51	33.74
slToc159-1	Solyc09074940.1.1	9	–	1–4212	–	4212 (1403)	4.51	151.06
slToc159-2	XM_004229523	1	–	1–3309	3310–3462	3489(1162)	4.80	127.52
slToc159-3	Solyc11g043010.1.1	11	–	1–522,617–1246,1333–1504,1562–1765,1922–2428,2481–2636	–	2187 (728)	5.33	81.30
slToc159-4	XM_004230964	1	–	1–4098	4099–4324	4101 (1366)	4.75	149.78

a Chromosomal localization of the tomato Toc GTPase genes.

b Domain of Toc GTPase genes in 5′UTR.

c Domains of Toc GTPase genes’s exton.

d Domain of Toc GTPase genes in 3′UTR.

e Length of open reading frame in base pairs and the number of amino acids of the deduced polypeptide in brackets.

f Isoelectric point (pI) of the deduced polypeptide.

g Molecular weight (kilodaltons) of the deduced polypeptide.

–: Not included.

### Sequence Analysis

The Toc159 and Toc34 families of GTPases share highly conserved GTP-binding domains, and have been characterized as being receptors in *Arabidopsis* and pea [5], [13], [14], [21], [22], [28], [33], [34]. The members of the Toc159 receptor protein family contain three defined domains: a C-terminal membrane anchor domain (M-domain), a central GTPase domain (G-domain), and an N-terminal acidic domain (A-domain) (Fig. 1A). The G-domain (Fig. 1A, grey) and M-domains (Fig. 1A, black) are highly conserved, whereas the A-domains (Fig. 1A, white) vary considerably in sequence and length among homologues within and between species [21].

The deduced amino acid sequences of the members of the slToc159 family (Fig. 1B-1E) were compared to the members of the *Arabidopsis* Toc159 protein family. slToc159-2 exhibits 33.2% overall identity with atToc159 (including the A-, G- and M-domains), most of which can be attributed to similarities within the G- and M-domain (54.3% identity in the G-domain; 64.6% identity in the M-domains); slToc159-2 also shows 31.0% overall identity with atToc120 (40.0% identity in the G-domain, 41.2% identity in the M-domains), 30.0% overall identity with atToc132 (40.5% identity in the G-domain; 45.6% identity in the M-domains), and 31.4% overall identity with atToc90 (37.7% identity in the G-domain, 32.2% identity in the M-domains). Furthermore, it shares 37.5% overall identity with slToc159-1 (54.8% identity in the G-domain; 61.0% identity in the M-domains), 28.2% overall identity with slToc159-3 (33.3% identity in the G-domain; 25.7% identity in the M-domains) and 27.7% overall identity with slToc159-4 (33.9% identity in the G-domain; 34.0% identity in the M-domains) (Fig. 1B). The similarities of the three other putative tomato Toc159 homologues are also reported in Figure 1. Overall, slToc159-1 ranges from 21.2% identical (atToc90) to 47.1% identical (atToc159) to Arabidopsis Toc159 homologues (Fig. 1C), slToc159-3 ranges from 20.0% identical (atToc159) to 41.4% identical (atToc120) to the Arabidopsis Toc159 homologues (Fig. 1D) and slToc159-4 ranges from 23.3% identical (atToc90) to 48.7% identical (atToc120) to Arabidopsis Toc159 homologues (Fig. 1E).

Toc34, another GTP-binding protein of the chloroplast import apparatus, has been reported to play a regulatory function and serve as a receptor in import [18], [22], [38], [39]. In *Arabidopsis*, this subunit has two homologues, named atToc34 and atToc33 based on their predicted molecular masses [24]. Therefore, the deduced amino acid sequences of the two tomato Toc34 homologues were designated as slToc34-1 and slToc34-2 (Fig. 2). slToc34-1 and slToc34-2 show 64.8% sequence identity to each other, and exhibit approximately 60.1% and 64.6% sequence identity, respectively, compared to pea Toc34 (psToc34) (Fig. 2B and C); they show 53.3% identity and 55.9% identity with atToc33, respectively; and 56.3% identity and 58.2% identity with atToc34, respectively (Fig. 2B and C).

### Gene Structure Analysis and Chromosomal Location of Tomato Toc GTPases

**Figure 3:**
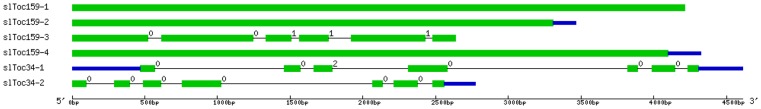
Exon-intron organization of tomato Toc GTPase genes. Exons and introns were predicted as described in the Materials and Methods. Exons are represented by green boxes, introns by black lines; blue lines correspond to the 5′ UTR and 3′UTR.

The cDNA sequences of the tomato Toc GTPases were compared to the *Solanum lycopersicum* genome (http://mips.helmholtz-muenchen.de/plant/tomato/searchjsp/index.jsp) to determine the gene structure for each Toc GTPase gene (Table 2, Fig. 3). The analysis revealed that the genes for slToc34-1, slToc34-2, slToc159-1, slToc159-2, Toc159-3 and slToc159-4 are located on chromosome numbers 3, 5, 9, 1, 11 and 1 respectively (Table 2). The gene structures for slToc159-1, slToc159-2 and slToc159-4 are similar: the coding regions for these three proteins are long, including exons of 4212 bp (slToc159-1), 3489 bp (slToc159-2) and 4101 bp (slToc159-4), respectively. None of these three genes contain any introns. In comparison, the gene encoding slToc159-3 is relatively complicated, comprising 6 exons and 5 introns. This diversity of gene structure within the Toc159 gene family has also been reported for the comparable components of the import complex from Arabidopsis [40]. The genes encoding slToc34-1 and slToc34-2 each have seven exons: the last five of these exons are nearly same size for both genes. In Arabidopsis, the genes encoding atToc34 and atToc33 each consist of six introns and seven exons; and these two genes also contain five exons that are exactly the same size [40]. These results provide evidence for a gene duplication event within the Toc34 family. The similarity in gene structure suggests that these two coding regions have diverged from one to another only relatively recently after the duplication of a common ancestral gene.

### Motifs of the Tomato Toc GTPases

**Figure 4:**
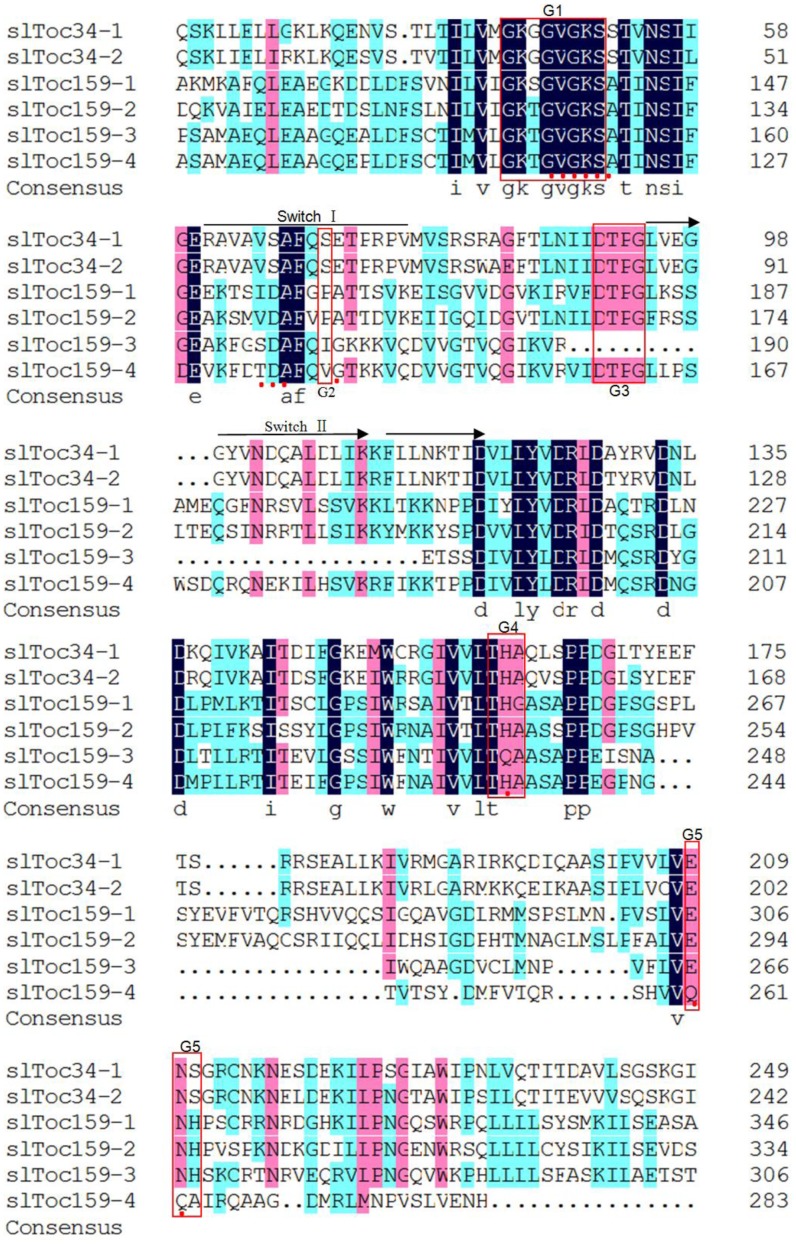
Conserved features of the G-domains of the tomato Toc GTPases. Red dots indicate residues corresponding to the GTP/Mg^2+^ binding site; arrows show Switch I and Switch II regions; red boxes represent the G1-G5 motifs. Gaps to optimize alignments are designated by dots. The consensus amino acids among all five sequences are indicated by black color. Amino acids are numbered on the right side of the sequence.

To gain additional insight into the six tomato Toc GTPase cDNAs that were identified, the features of the deduced amino acid sequences were analyzed in more detail using the NCBI Conserved Domain Database (http://www.ncbi.nlm.nih.gov/Structure/cdd/wrpsb.cgi). The analysis confirmed that these proteins are GTPases, as many well-characterized GTPase motifs were identified (Fig. 4). The specific motifs that were identified include the 13-residue GTP/Mg^2+^ binding site; Switch I and Switch II regions, which form two surface loops that undergo conformational changes upon GTP binding [41]; a G1 box motif (GXXXXGK[T/S]), also known as the P-loop or the Walker A motif [41]; a G2 box motif (which overlaps with the Switch I region) in which only Thr is conserved throughout the superfamily, although surrounding residues are conserved within families [41]; the G3 box motif (DXXG), which overlaps the Switch II region, and includes the Walker B motif [41]; a G4 box motif ([N/T]KXD); and a G5 box motif ([C/S]A[K/L/T]) [41].

### Phylogenetic Analysis

**Figure 5:**
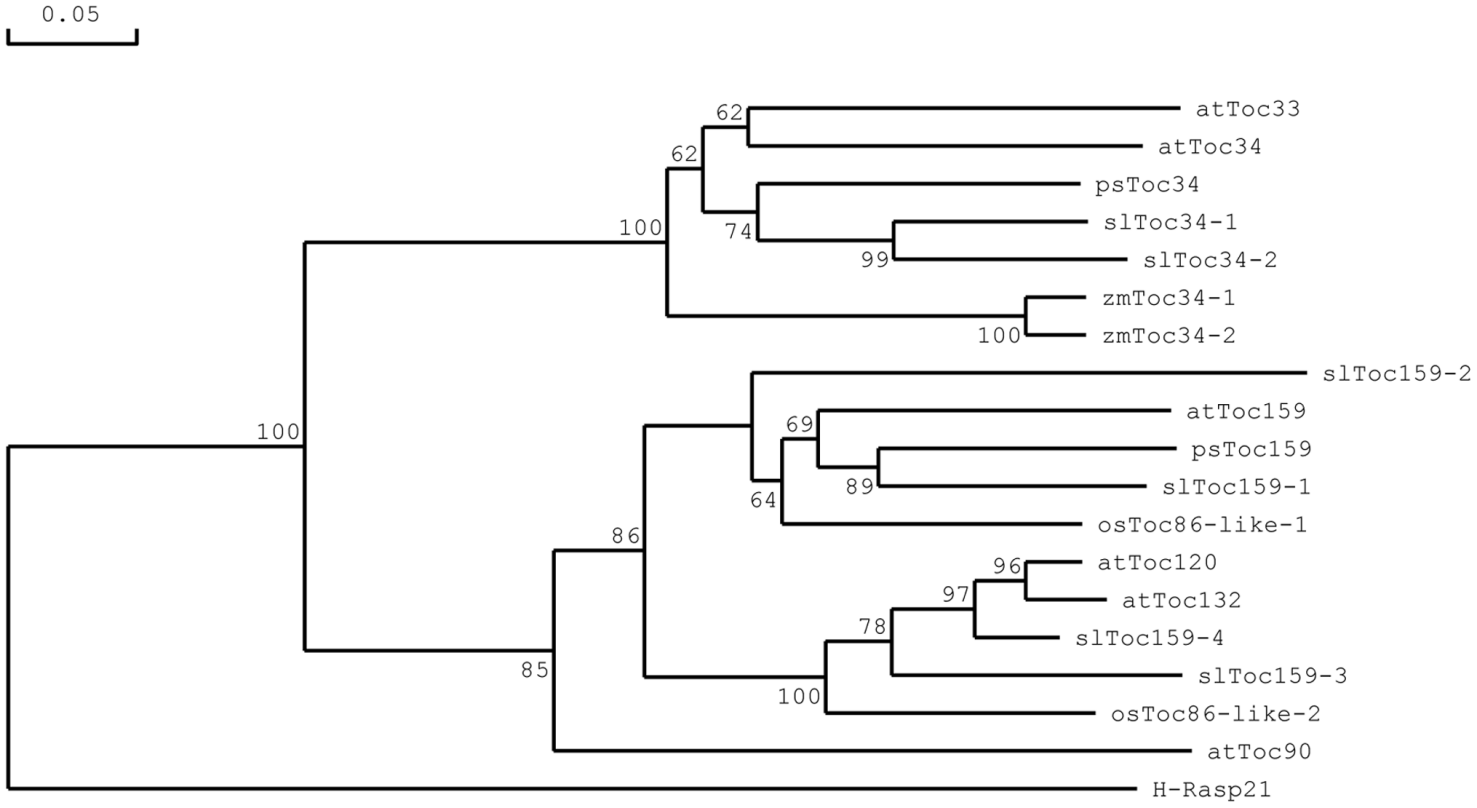
Phylogenetic Analysis of Toc-GTPases from Tomato and Other Species. Amino acid sequences of Toc159 and Toc34 homologues from different species were aligned and used to produce a phylogenetic tree. Bootstrap values are given in the branch. Genes and accession numbers of the sequences used are as follows: slToc34-1, XM_004235160; slToc34-2, Solyc05g052160.2.1; slToc159-1, Solyc09074940.1.1; slToc159-2, XM_004229523; slToc159-3, Solyc11g043010.1.1; slToc159-4, XM_004230964; atToc159, At4g02510; atToc132, At2g16640; atToc120, At3g16620; atToc90, At5g20300; atToc33, At1g02280; atToc34, At5g05000; psToc159, AAF75761; psToc34, Q41009; osToc86-like_1, AAG48839; osToc86-like_2, AAK43509; zmToc34-1, CAB65537; zmToc34-2,CAB77551; H-Ras p21, P01112. Species of origin of the sequencesare indicated as follows: sl, *Solanum lycopersicon* at, *Arabidopsis thaliana*; ps, *Pisum sativum*; os, *Oryza sativa*; zm, *Zea mays*. H-Ras p21 is from human.

To evaluate the evolutionary relationships of the different Toc GTPases from tomato, a phylogenetic analysis was conducted using Toc GTPase homologues from several different species. For this analysis, the Toc159- and Toc34-related sequences from tomato were compared to sequences from *Arabidopsis* and pea, the monocotyledonous species rice (*Oryza sativa*) and maize (*Zea mays*), as well as a distantly related GTPase from human, H-Ras p21, which was used as an outgroup. The analysis produced a phylogenetic tree that contained two main groups, the Toc159-related proteins and Toc34-related proteins (Fig. 5), and the clustering of the tomato Toc GTPases within those groups confirmed that the designated names were appropriate.

In the Toc34-related protein group, slToc34-1 and slToc34-2 are within the same clade, which was supported by a bootstrap value of 99%, with psToc34 from the dicotyledonous species, pea; the *Arabidopsis* proteins atToc33 and atToc34 also group together (Fig. 5). The Toc34 proteins from the monocotyledonous species *Zea mays* form a separate subclade (Fig. 5).

In the Toc159-related protein group there are two distinct subfamilies: the atToc159-like and atToc132/atToc120-like groups. Tomato slToc159-1 and slToc159-2, group together in the atToc159-like subgroup with *Arabidopsis* atToc159, psToc159 and the osToc86-like-1 protein from rice. slToc159-1 and psToc159 are within the same clade with 89% bootstrap values. The atToc132/atToc120-like subgroup is comprised of tomato slToc159-3, slToc159-4, *Arabidopsis* atToc132 and atToc120, and one of the rice osToc86-like-2 proteins. Here, slToc159-3 is more distantly related to atToc132/atToc120 than is slToc159-4. Although atToc90 lacks an A-domain, it clearly belongs to the Toc159 family. slToc159-3, is more distantly related to the Toc159 sub-group, and not closely related to atToc90, suggesting that these proteins may not share a close functional relationship either.

Collectively, these data show that the Toc GTPases in tomato are similar to those in other higher plants studied to date, in that they have two fundamentally different groups, a Toc159-related group, which can be further sub-divided into atToc159-like and atToc132/120-like sub-groups, and a Toc34-related group. Applying a cutoff value of 50%, all clades were supported by bootstrap analysis.

### Expression Profiles of the TocGTPase Genes in Tomato

Differential expression patterns corresponding to different functions in various plant tissues have been reported for several of the Toc GTPases [12], [14], [24]. The expression patterns of Toc GTPase-related proteins slToc34-1, slToc34-2, slToc159-1, slToc159-2, slToc159-3 and slToc159-4 from tomato were examined with the goal of providing insight into potential functional differences. A quantitative Real-Time PCR approach was used to determine the relative expression of the tomato Toc GTPases in a variety of tissues and at different stages of development. The expression of representative photosynthetic (small subunit of Rubisco, RbcS) and non-photosynthetic (E1α subunit of pyruvate dehydrogenase, pE1α) genes for plastid proteins that are encoded in the nucleus was also examined, for comparison.

**Figure 6:**
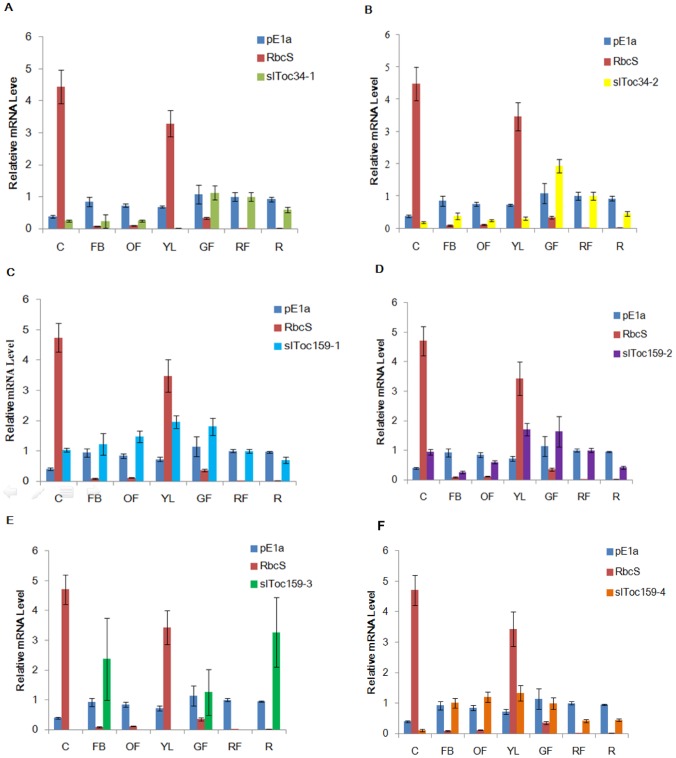
Relative gene expression of TocGTPase genes in different tomato organs analyzed by QRT-PCR. (A) Relative mRNA expression level of slToc34-1. (B) Relative mRNA expression level of slToc34-2. (C) Relative mRNA expression level of slToc159-1. (D) Relative mRNA expression level of slToc159-2. (E) Relative mRNA expression level of slToc159-3. (F) Relative mRNA expression level of slToc159-4. (Cotyledons, C; Flower Buds, FB; Opening Flowers, OF; Young Leaves, GL; Green Fruit, GF; Red Fruit, RF; Roots, R). Y-axes are scales of relative expression level. (error bars indicate ±SD).

As expected, the RbcS gene was found to be highly expressed in cotyledons and young leaves, and at much lower levels in mature and non-photosynthetic tissues, with nearly no expression detected in root and red fruit (Fig. 6). In contrast, the pE1α gene showed relatively uniform levels of expression in all tested tissues, although relatively lower expression levels were detected in cotyledons (Fig. 6).

Expression levels of the slToc34 family were generally lower than that of the slToc159 family in most tissues (Fig. 6). The expression of slToc34-1 was highest in green fruit, root and red fruit as compared to young, photosynthetic tissues and was lowest in young leaves (Fig. 6A). slToc34-2 had a similar expression pattern as slToc34-1; it was expressed most highly in green fruit and red fruit, and at much lower levels in cotyledons, flower buds, opening flowers, young leaves and at a relatively moderate level in roots (Fig. 6B). These data suggest that slToc34-1 and slToc34-2 are generally more highly expressed in non-photosynthetic tissues. On the other hand, expression levels of slToc159-1 is highest in green, photosynthetic tissues such as young leaves, green fruit, flower buds and opening flowers, at lower levels in roots, and moderate levels in cotyledons and red fruit (Fig. 6C). This pattern is similar to what has been reported for atToc159 in Arabidopsis [12]. The highest relative expression of slToc159-2 was observed in young leaves and green fruit (Fig. 6D). Interestingly, the relative expression levels of slToc159-1 in flower buds and opening flowers is higher than slToc159-2 (Fig. 6C and D), suggesting that slToc159-1 might be more important than slToc159-2 for import into plastids in flower tissues.

slToc159-3 is distinct from the other tomato Toc159 GTPases (Fig. 6E). It displayed its highest expression levels in root, flower buds and green fruit tissues. Interestingly, this transcript could not be detected in the other tissues (cotyledons, opening flowers, young leaves and red fruit) tested in this study. This suggests that the expression of slToc159-3 may be restricted to specific plastid types or that it is only needed at specific times or under specific conditions.

We found that slToc159-4 had an expression pattern similar to slToc159-1 (Fig. 6F); it also showed the highest expression levels in flower buds, open flowers, green fruit and young leaves; moderate levels in red fruit and roots; and the lowest level of expression in cotyledons.

It was also noted that the expression levels of all Toc GTPase genes from tomato were generally higher in green fruit as compared to other tissues. This is noteworthy, because the plastids in this tissue are undergoing re-differentiation from green plastids (chloroplasts) to non-green plastids (chromoplasts), which requires the import of a different complement of nucleus-encoded proteins. The high expression levels of the Toc GTPases in green fruit tissue may reflect the changing physiology of ripening fruit and the associated changes in preprotein import that would accompany plastid re-differentiation.

## Discussion

Most studies of components of the chloroplast protein import apparatus have focused on the model plant Arabidopsis. For many of the Toc components, multiple homologues can be found within the Arabidopsis genome [11], [12], [24], [28], [37], [40], [42], [43]. The recent completion of the tomato genome [31] has provided the opportunity to determine whether multigene families of Toc components also exist in tomato. As tomato also provides abundant sources of both photosynthetic (chloroplasts) and non-photosynthetic (especially chromoplasts) plastids, comparing the expression patterns of the Toc GTPases is a first step toward a more thorough understanding of how the various homologues of these gene families are potentially involved in the import of different classes of nucleus-encoded plastid proteins and the biogenesis of different plastid types. In this study, the Toc GTPase genes in tomato have been examined for the first time, and have been designated as slToc34-1 (XM_004235160), slToc34-2 (Solyc05g052160.2.1), slToc159-1 (Solyc09g074940.1.1), slToc159-2 (XM_004229523), slToc159-3 (Solyc11g043010.1.1) and slToc159-4 (XM_004230964) based on their predicted molecular masses, a phylogenetic tree analysis, and in accordance with the accepted nomenclature for the naming of proteins associated with chloroplast protein import [33], to lay a foundation for future functional studies of these Toc complex components in tomato. Several of the cDNAs representing these genes were cloned, and the expression profiles of all Toc GTPases were also examined, as a starting point for future studies on the functional differences among the tomato Toc GTPase homologues.

Our analysis of the tomato genome identified five putative Toc GTPases, as did an automated gene prediction analysis using the NCBI: Gnomon method, which is supported by mRNA and EST evidence [36]. Based on a multiple sequence alignment (Fig. S1, S2, S3, S4, and S5) of the 5 cDNAs originally identified in the present analysis and those identified in the NCBI analysis, we concluded that four of the cDNAs (slToc34-1, slToc34-2, slToc159-1 and slToc159-2) identified in the two analyses correspond to the same genes, even though the sequence of the cDNAs for only one (slToc34-2) of these four was identical in both of the analyses. In the three cases where there were minor differences in the sequences of the cDNAs identified in the two analyses, it was decided that the longer sequence would be used for the purpose of this study. It remains possible that the different cDNAs represent splicing or other variants, but an investigation into this possibility was outside the scope of the present study.

In the case of slToc34-1 (XM_004235160), the additional 126-bp in the NCBI-derived sequence corresponded to an upstream exon which is consistently present in every other known Toc34 homologue that has been reported from all species. In the case of slToc159-2 (XM_004229523), the additional 180-bp present in the NCBI-reported sequence is in N-terminus (A-domain), and in the case of slToc159-1 (Solyc09074940.1.1), the extra 384-bp in the sequence identified in the present analysis was also in N-terminus (A-domain).

The coding sequences for slToc159-3 (Solyc11g043010.1.1) and slToc159-4 (XM_004230964) share only 36.0% amino acid identity, and the genes are located on chromosome numbers 11 and 1, respectively. It was therefore concluded that these do not correspond to the same gene. Interestingly, slToc159-3 was not identified in the NCBI-reported gene prediction analysis; it is not clear why this gene was missed, nor is it clear why slToc159-4 was identified in the present genome analysis, but not using the NCBI gene prediction method. We included both of these genes in our bioinformatics analysis. In total, six Toc GTPases (slToc34-1, slToc34-2, slToc159-1, slToc159-2, slToc159-3 and slToc159-4) were analyzed.

All tomato Toc GTPases that were identified in this study have a high sequence similarity with the comparable proteins from Arabidopsis (Fig. 1 and Fig. 2), suggesting that these proteins are also components of the plastid protein import apparatus in tomato and that the protein import system is conserved in this species, making tomato a valid model for the study of protein import.

The conserved domain analysis of the Toc GTPase import components indicated that the Toc34 and Toc159 family proteins belong to a unique class of GTPases that were first identified in pea as being involved in preprotein recognition and binding [18], [20], [45]. Indeed, there are eight conserved GTPase motifs (GTP-binding site, Switch I and Switch II domains, and G1-G5 boxes) in slToc34-1, slToc34-2, slToc159-1, slToc159-2, slToc159-3 and slToc159-4 (Fig. 4). In addition, Toc159 proteins have three domains: an intrinsically disordered N-terminal acidic (A) domain, which is very sensitive to proteolysis, giving rise to an 86-kDa fragment [16], [18], [44], [45]; a central GTPase (G) domain related to the Toc34 G-domain; and, a hydrophilic M-domain that anchors the proteins in the outer chloroplast membrane through an unknown mechanism [15], [17] (Fig. 1A). However, sequence similarities vary between the domains of the Toc159 family members, with the G- and M-domains displaying significantly higher sequence conservation than the A-domain [14]. Similarly conserved domains between different species suggest high conservation of these import components in all species.

The phylogenetic analyses in the present study (Fig. 5) were consistent with the notion that all Toc GTPases fall into two subgroups, homologues of Toc159 and homologues of Toc34. slToc34-1 and slToc34-2 cluster with all the Toc34-related proteins. slToc159-1 and slToc159-2 segregate with a sub-group of Toc159-related proteins, that could be called the atToc159-like sub-group. slToc159-3 and slToc159-4, on the other hand, cluster more with another sub-group of Toc159-related proteins that include atToc132 and atToc120. Thus, subsequent reference is made to at the atToc132/120-like subgroup. Based on the results of the phylogenetic analysis, as well as the sequence identities reported in Figure 1, it is possible that slToc159-3 and slToc159-4 should be renamed slToc132/120-1 and slToc132/120-2 to indicate the differences. Although a similar phylogenetic study was carried out by Hiltbrunner et al. [17], the reliability of the predicted clades was not tested by bootstrap analysis.

In order to begin to address the roles of the Toc34 family and the Toc159 family in the development of tomato and plastid biogenesis, an expression analysis was performed by quantitative real-time PCR. In general, the data presented here indicate that slToc34-1 and slToc34-2 were expressed at higher levels in non-photosynthetic tissues, whereas expression levels of slToc159-1 and slToc159-4 were highest in photosynthetic tissues (Fig. 6), and expression levels of slToc159-2 was not significantly different between photosynthetic and non-photosynthetic tissues. slToc159-3 had the most distinct expression pattern, with notable expression levels only detected in flower buds, green fruit and roots, suggesting that it might have the most specialized role of all the slToc159 isoforms. On the basis of the results of this study and those of previous studies, it is proposed that the Toc159 and Toc34 homologues in tomato combine to form distinct Toc complexes, as they are thought to do in other species. The possibility that these distinct complexes have functionally distinct roles in the recognition and import of different classes of preproteins, and therefore in the biogenesis of different plastid types, can now be investigated. However, additional work is required to definitively determine the physiological role of the distinct Toc GTPases and the distinct translocons that they appear to comprise. It should also be noted that we have only compared the transcript levels of the Toc GTPases; It will also be important to determine the relative levels of the corresponding protein.

The observations reported here support the possibility that there are multiple types of Toc GTPases that assemble into structurally distinct Toc complexes at the surface of tomato plastids, as has been hypothesized in Arabidopsis; furthermore, it is possible that such distinct Toc complexes are also functionally distinct, each facilitating the import of a particular subset of precursor proteins. However, because the stoichiometry of the subunits within the outer membrane translocons is not known, such questions cannot be answered in this study. More detailed genetic and biochemical analyses are required to address this possibility in more detail.

In the present work, the Toc GTPase cDNAs from tomato were identified, cloned and analyzed, constituting the first report on the chloroplast protein import apparatus for this species. The results revealed that tomato Toc159 and Toc34 homologues share high sequence similarity with the comparable import apparatus components from the Arabidopsis and pea. This suggests that the plastid protein import system is conserved in tomato, making tomato a potentially new and interesting model system, especially for biochemical studies aimed at elucidating the differences in import between chloroplasts and other plastid types, given the abundance of non-photosynthetic plastids such as chromoplasts in ripe fruit. This work also demonstrates that slToc34-1 and slToc34-2 are generally more highly expressed in non-photosynthetic tissues. slToc159-1 and slToc159-4 were expressed at higher levels in photosynthetic tissues, and expression level of slToc159-2 was not significantly different in photosynthetic or non-photosynthetic tissues, and that slToc159-3 is primarily expressed in flower buds, green fruit and roots. It is possible that slToc159-1 and/or slToc159-4 are the functional orthologues of atToc159, and that slToc159-2 and/or slToc159-3 are the functional orthologues of atToc132 and atToc120. Collectively, the data lay a foundation for future functional studies of these Toc complex components in tomato.

## Supporting Information

Figure S1 **Multiple sequence alignment of the putative amino acid of slToc34-1 (Solyc03g095220.2.1) and slToc34-like-1 (XM_004235160.1).** Gaps to optimize alignments are designated by dots. The consensus amino acid identity between two protein is indicated by black color. Amino acids are numbered on the right side of the sequence.
(TIF)

Figure S2 **Multiple sequence alignment of the putative amino acid of slToc34-2 (Solyc05g052160.2.1) and slToc34-like-2 (XM_004239929.1).** Gaps to optimize alignments are designated by dots. The consensus amino acid identity between two protein is indicated by black color. Amino acids are numbered on the right side of the sequence.
(TIF)

Figure S3 **Multiple sequence alignment of the putative amino acid of slToc159-1 (Solyc09074940.1.1) and slToc159-like-1 (XM_004247489).** Gaps to optimize alignments are designated by dots. The consensus amino acid identity between two protein is indicated by black color. Amino acids are numbered on the right side of the sequence.
(TIF)

Figure S4 **Multiple sequence alignment of the putative amino acid of slToc159-2 (Solyc01g080780.2.1) and slToc159-like-2 (XM_004229523).** Gaps to optimize alignments are designated by dots. The consensus amino acid identity between two protein is indicated by black color. Amino acids are numbered on the right side of the sequence.
(TIF)

Figure S5 **Multiple sequence alignment of the putative amino acid of slToc159-3 (Solyc11g043010.1.1) and slToc159-like-3 (XM_004230964.1).** Gaps to optimize alignments are designated by dots. The consensus amino acid identity between two protein is indicated by black color. Amino acids are numbered on the right side of the sequence.
(TIF)

Data S1 **The cDNA and amino acid sequences of each our identified tomato Toc GTPase.**
(DOCX)

Data S2 **The amino acid sequences of each our identified tomato Toc GTPase.**
(DOCX)

Data S3 **The cDNA sequences of each NCBI identified tomato Toc GTPase.**
(DOCX)

Data S4 **The amino acid sequences of each NCBI identified tomato Toc GTPase.**
(DOCX)
