# Supplementary figures and images for: Molecular Characterization and Expression Analysis of Chloroplast Protein Import Components in Tomato (Solanum lycopersicum)
Source: PLoS One. 2014 Apr 21;9(4):e95088. doi: 10.1371/journal.pone.0095088 (PMC3994019; doi:10.1371/journal.pone.0095088)

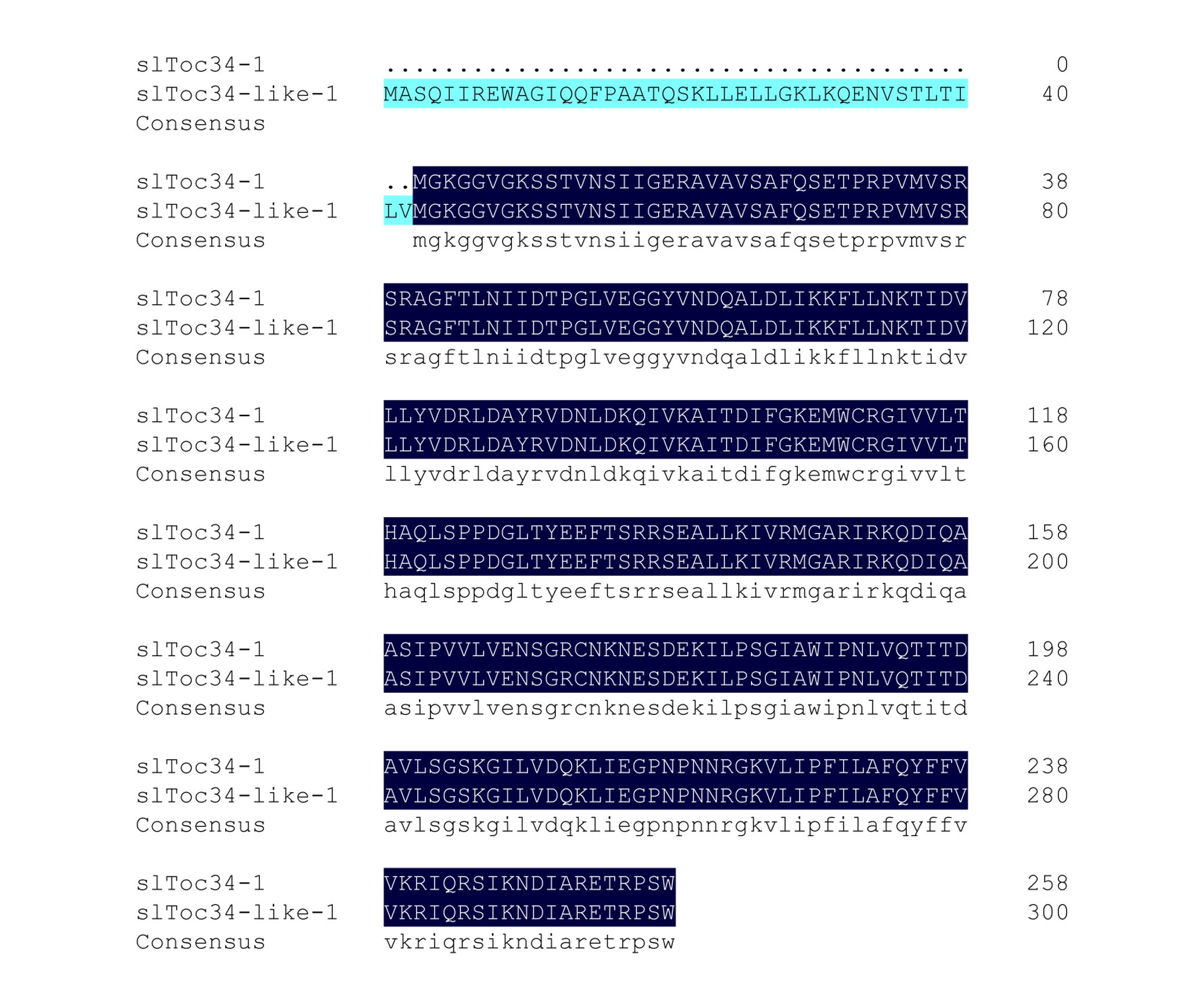

Supplement: Figure S1 — Multiple sequence alignment of the putative amino acid of slToc34-1 (Solyc03g095220.2.1) and slToc34-like-1 (XM_004235160.1). Gaps to optimize alignments are designated by dots. The consensus amino acid identity between two protein is indicated by black color. Amino acids are numbered on the right side of the sequence. (TIF) [file pone.0095088.s001.tif]

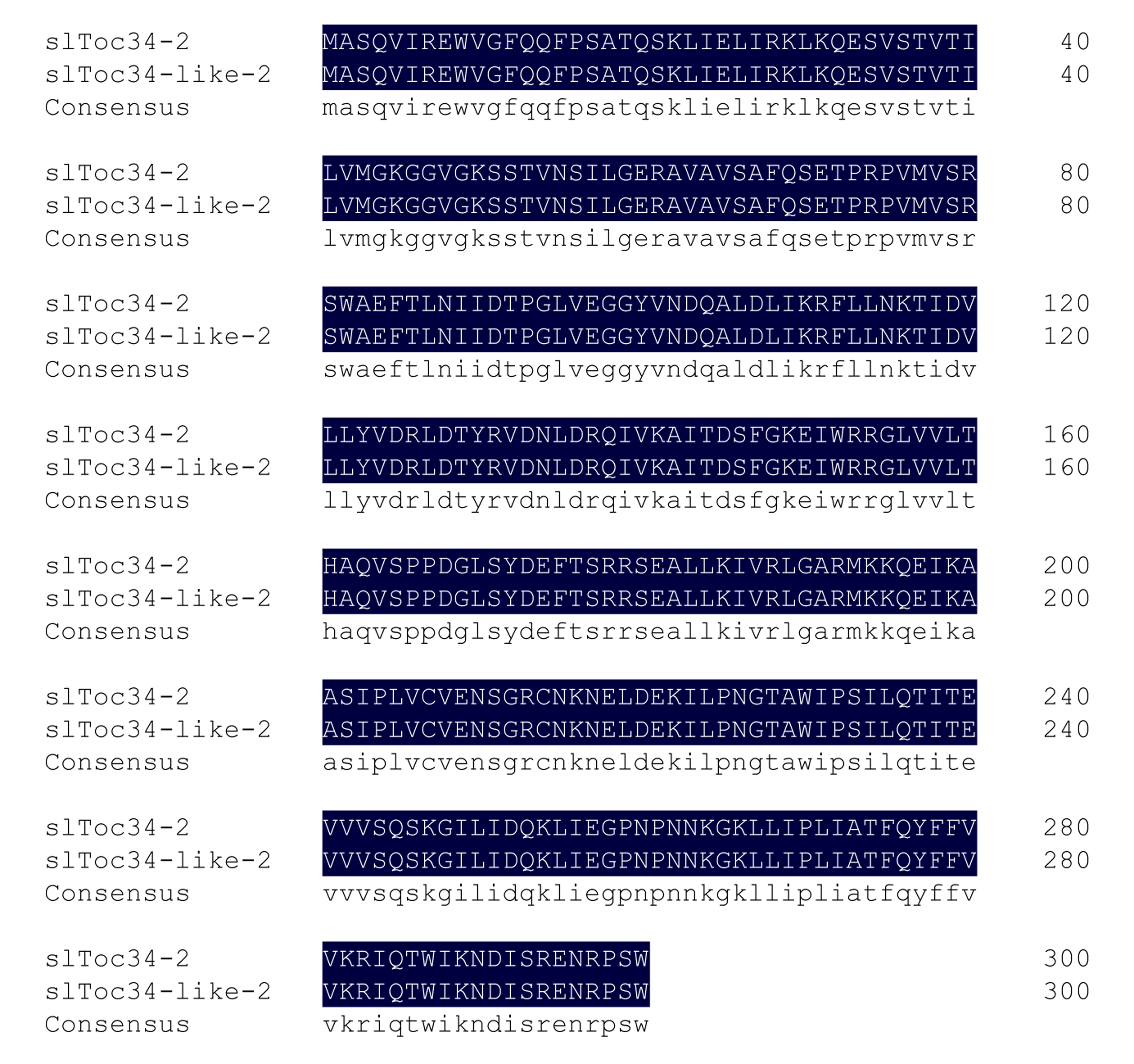

Supplement: Figure S2 — Multiple sequence alignment of the putative amino acid of slToc34-2 (Solyc05g052160.2.1) and slToc34-like-2 (XM_004239929.1). Gaps to optimize alignments are designated by dots. The consensus amino acid identity between two protein is indicated by black color. Amino acids are numbered on the right side of the sequence. (TIF) [file pone.0095088.s002.tif]

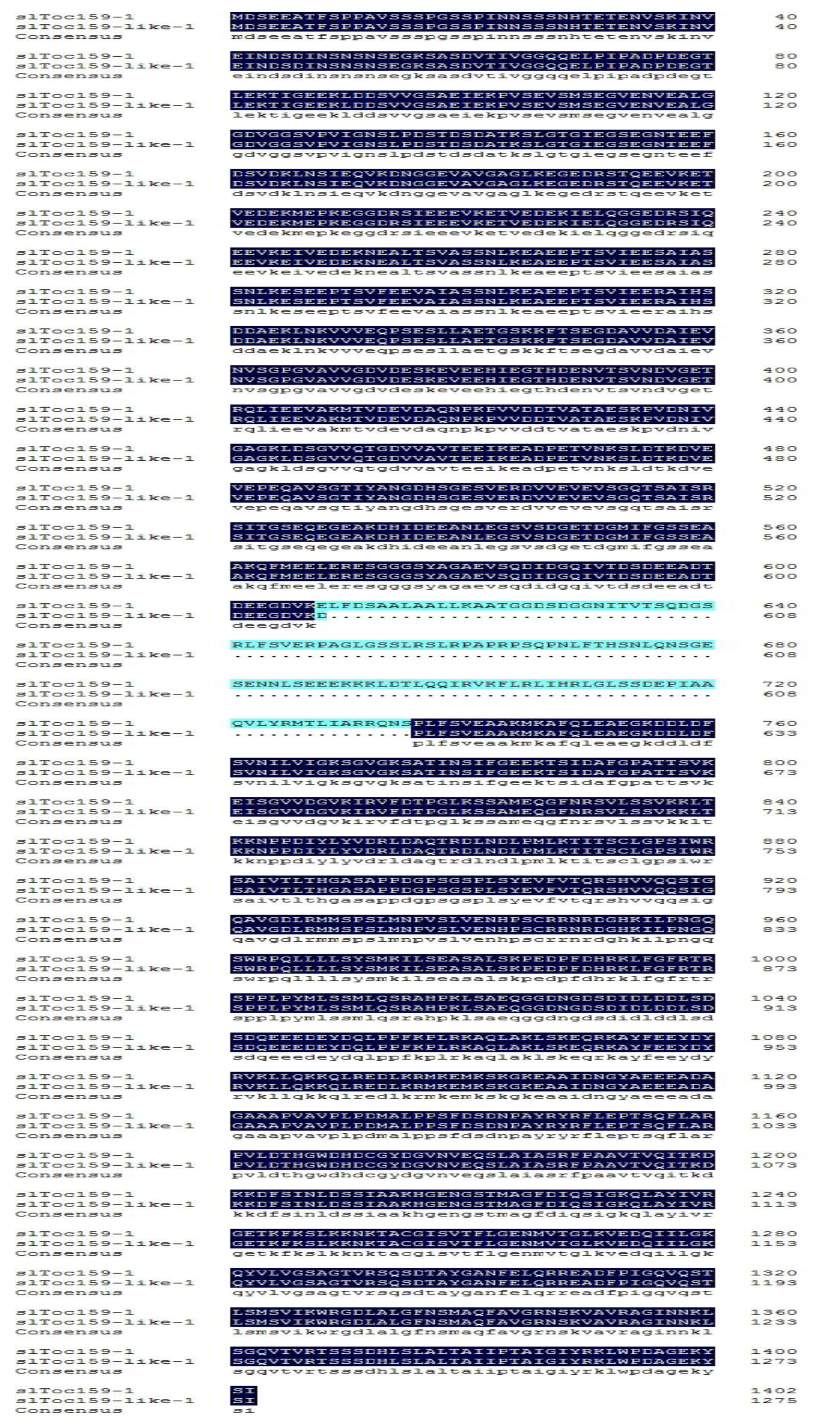

Supplement: Figure S3 — Multiple sequence alignment of the putative amino acid of slToc159-1 (Solyc09074940.1.1) and slToc159-like-1 (XM_004247489). Gaps to optimize alignments are designated by dots. The consensus amino acid identity between two protein is indicated by black color. Amino acids are numbered on the right side of the sequence. (TIF) [file pone.0095088.s003.tif]

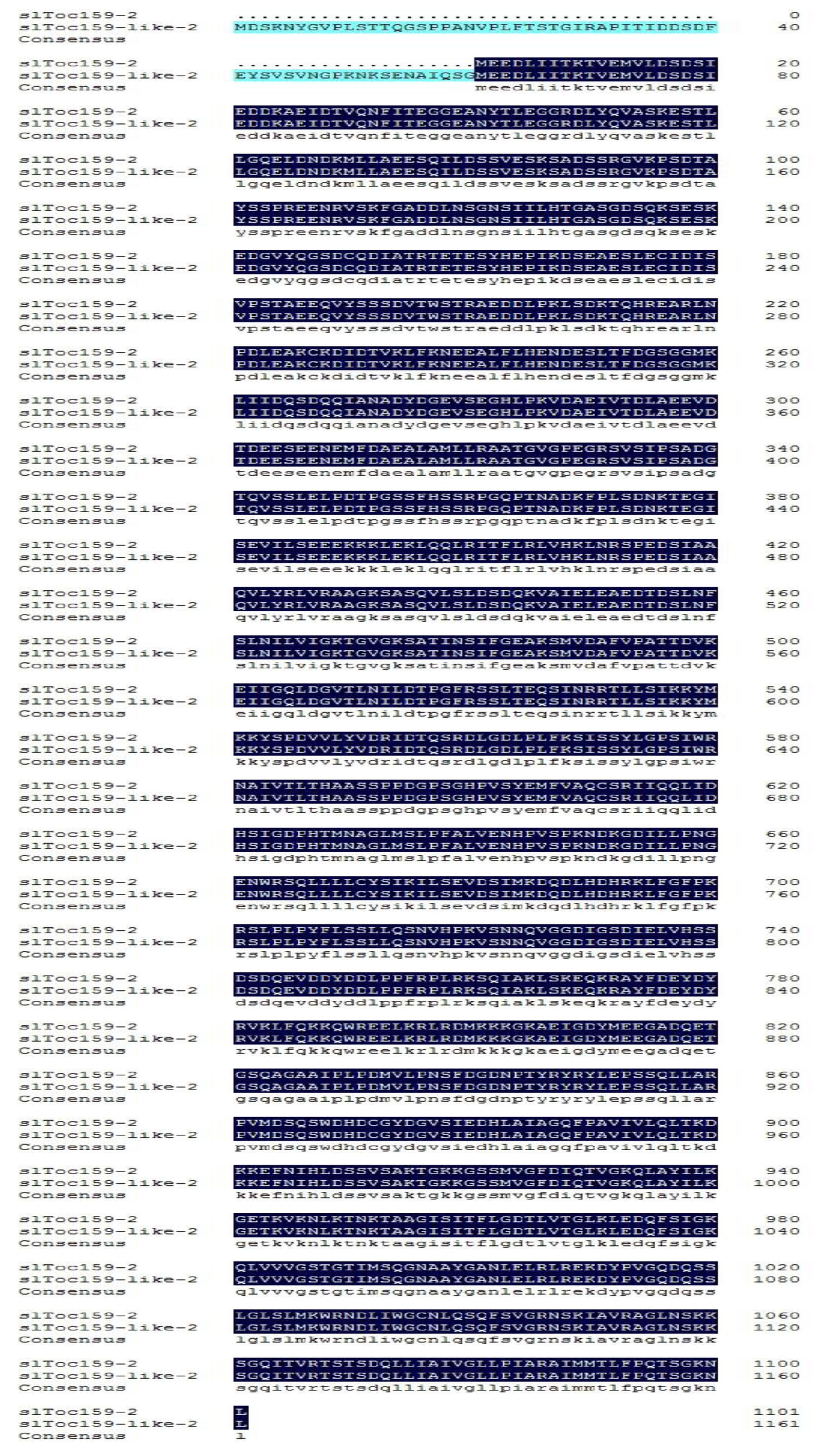

Supplement: Figure S4 — Multiple sequence alignment of the putative amino acid of slToc159-2 (Solyc01g080780.2.1) and slToc159-like-2 (XM_004229523). Gaps to optimize alignments are designated by dots. The consensus amino acid identity between two protein is indicated by black color. Amino acids are numbered on the right side of the sequence. (TIF) [file pone.0095088.s004.tif]

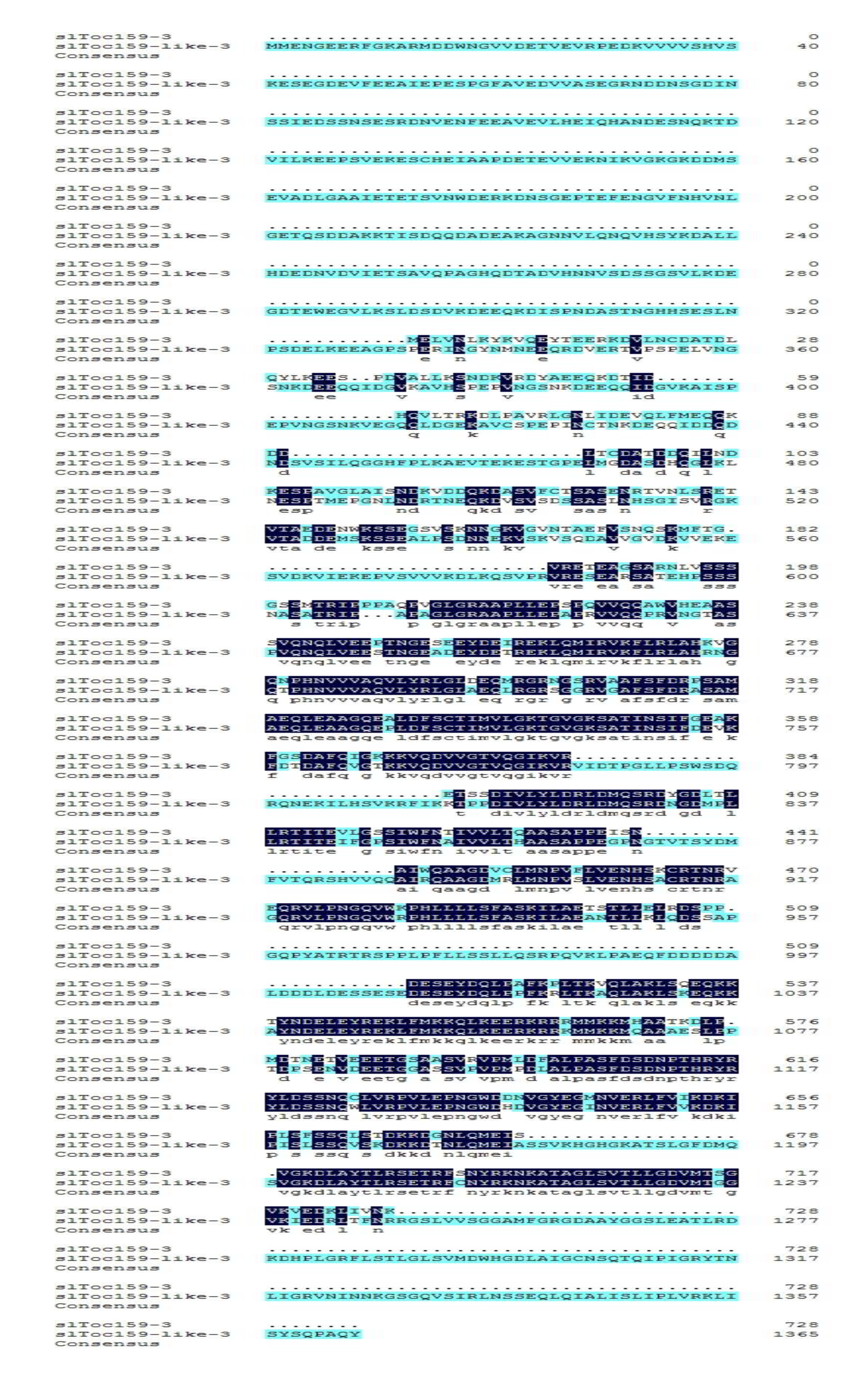

Supplement: Figure S5 — Multiple sequence alignment of the putative amino acid of slToc159-3 (Solyc11g043010.1.1) and slToc159-like-3 (XM_004230964.1). Gaps to optimize alignments are designated by dots. The consensus amino acid identity between two protein is indicated by black color. Amino acids are numbered on the right side of the sequence. (TIF) [file pone.0095088.s005.tif]
