# Supplementary material for: Molecular Characterization and Expression Analysis of Chloroplast Protein Import Components in Tomato (Solanum lycopersicum)
Source: PLoS One. 2014 Apr 21;9(4):e95088. doi: 10.1371/journal.pone.0095088 (PMC3994019; doi:10.1371/journal.pone.0095088)
Supplement: Data S1 — The cDNA and amino acid sequences of each our identified tomato Toc GTPase. (DOCX) [file pone.0095088.s006.docx]

>slToc34-1 (Solyc03g095220.2.1)

ATGGGGAAAGGCGGTGTTGGAAAATCATCAACGGTGAACTCAATTATTGGGGAAAGAGCAGTTGCTGTTAGTGCATTTCAGTCAGAAACTCCCAGACCGGTGATGGTTTCGCGTTCACGAGCAGGTTTTACACTAAACATAATTGACACCCCAGGGCTAGTTGAAGGAGGATACGTCAATGACCAGGCTCTTGATCTCATAAAGAAGTTCCTCTTGAACAAGACAATTGATGTTTTGCTATATGTGGATCGTCTGGATGCATATAGAGTGGATAACTTGGATAAGCAGATTGTGAAGGCTATTACTGATATTTTTGGCAAGGGAATGTGGTGCCGAGGAATTGTGGTTCTCACTCATGCTCAGCTTTCCCCTCCTGATGGATTGACTTATGAAGAATTCACGTCCCGAAGATCAGAGGCACTTTTGAAAATCGTCCGGATGGGTGCTCGAATCAGGAAACAAGACATTCAGGCTGCTTCAATTCCTGTTGTCTTGGTTGAGAATAGTGGCAGATGTAACAAGAATGAAAGTGACGAAAAGATTCTTCCAAGTGGAATTGCGTGGATACCCAATTTAGTCCAAACTATTACAGATGCTGTTTTAAGTGGAAGCAAGGGTATTTTGGTTGATCAAAAACTGATTGAAGGTCCTAATCCAAACAATAGGGGCAAAGTGCTAATTCCTTTTATACTAGCATTCCAGTATTTCTTTGTAGTGAAAAGGATCCAGAGGTCCATTAAGAATGATATTGCACGGGAGACCAGGCCTTCATGGGCATAA

>slToc34-2 (Solyc05g052160.2.1)

ATGGCATCTCAGGTGATAAGAGAATGGGTTGGATTTCAGCAGTTTCCTTCAGCCACTCAGTCAAAGTTAATTGAATTGATAAGAAAATTGAAGCAGGAGAGTGTAAGTACAGTGACAATCCTAGTAATGGGGAAAGGTGGTGTTGGAAAATCTTCAACTGTAAACTCAATTTTAGGGGAAAGAGCAGTTGCTGTCAGTGCGTTTCAGTCAGAAACTCCAAGACCAGTGATGGTTTCACGTTCATGGGCAGAATTTACATTGAACATTATCGACACCCCCGGGCTGGTTGAAGGAGGATATGTCAATGACCAGGCTCTTGATCTCATAAAGAGGTTCCTCCTGAACAAGACAATTGATGTTTTGCTTTATGTGGATCGTCTTGATACATATAGGGTGGACAATTTGGATAGGCAGATTGTAAAGGCCATAACAGATAGTTTCGGCAAGGAAATATGGCGTAGAGGACTTGTGGTCCTCACACATGCTCAGGTCTCCCCTCCTGATGGATTGAGTTATGACGAGTTCACTTCGAGAAGATCAGAGGCACTTCTGAAAATTGTCCGCCTGGGAGCTCGAATGAAGAAACAAGAGATCAAGGCTGCTTCAATTCCTCTTGTTTGCGTTGAGAACAGTGGGAGATGTAACAAGAATGAACTCGACGAAAAGATTCTTCCAAATGGAACTGCTTGGATACCCAGTATACTCCAAACTATTACCGAAGTTGTCGTAAGTCAAAGCAAGGGTATCTTGATTGATCAAAAATTGATCGAAGGACCAAATCCCAACAATNAAGGGCAAATTGCTGATCCCTCTTATCGCAACATTCCAGTATTTCTTCGTTGTGAAAAGGATCCAGACGTGGNTCAAAAACGACATTTCAAGAGAGAACAGACCTTCATGGGCATAA

>slToc159-1 (Solyc09g074940.1.1)

ATGGATTCTGAAGAAGCGACGTTTTCGCCTCCTGCTGTTTCTTCTTCTCCAGGTTCTTCTCCCATCAACAATTCTTCTTCTAATCATACTGAAACTGAAAATGTCTCCAAAATTAATGTAGAAATCAATGATTCCGATATTAATAGTAATAGCAATAGTGAGGGTAAAAGCGCTAGTGATGTAACTATTGTGGGTGGTCAGCAAGAATTGCCAATTCCTGCTGACCCAGATGAGGGAACCCTAGAAAAAACTATTGGGGAAGAGAAGTTGGATGATTCCGTTGTGGGTTCTGCGGAAATTGAGAAGCCTGTTTCTGAGGTTTCTATGAGTGAGGGTGTTGAAAATGTTGAGGCTTTAGGGGGAGATGTTGGTGGTTCTGTTCCTGTTATTGGGAATAGTTTACCTGATTCTACTGACTCTGATGCTACCAAATCACTGGGGACGGGAATCGAGGGTTCGGAGGGTAATACTGAAGAGTTTGACTCGGTTGATAAGTTGAATTCAATTGAGCAGGTGAAAGATAATGGTGGTGAGGTTGCGGTTGGTGCAGGATTGAAAGAGGGTGAGGATAGGTCCACTCAGGAGGAGGTGAAGGAAACTGTGGAGGATGAAAAGATGGAACCGAAAGAGGGCGGGGATAGGTCTATCGAGGAGGAGGTGAAGGAGACTGTGGAGGATGAAAAGATAGAATTGCAAGGGGGTGAGGATAGGTCCATTCAGGAGGAGGTGAAGGAGATTGTGGAGGATGAAAAGAATGAAGCTTTAACTAGTGTTGCTTCATCTAATTTAAAGGAGGCTGAGGAACCTACCTCGGTCATCGAAGAGAGTGCTATTGCTTCATCCAATTTGAAAGAGTCTGAGGAACCTACCTCGGTCTTTGAAGAGGTTGCTATTGCTTCATCCAATTTGAAGGAGGCCGAAGAACCTACCTCGGTCATTGAAGAGAGAGCTATACATAGTGATGATGCCGAAAAACTCAATAAGGTGGTTGTTGAACAACCGTCGGAATCTTTGTTGGCTGAAACAGGTAGTAAGAAATTTACTTCCGAAGGAGATGCAGTTGTGGATGCTATTGAAGTCAATGTCTCAGGGCCAGGGGTTGCTGTTGTTGGAGATGTGGACGAGAGCAAGGAAGTGGAAGAACATATTGAAGGTACCCATGATGAAAATGTGACATCAGTAAATGATGTTGGTGAGACCAGACAACTTATTGAAGAAGTGGCTAAAATGACAGTTGATGAAGTAGATGCACAGAACCCTAAGCCTGTGGTGGATGATACTGTTGCAACTGCAGAATCAAAGCCTGTGGATAACATTGTTGGTGCTGGAAAACTTGATTCTGGAGTTGTTCAGACTGGTGATGTGGTAGCTGTTACTGAGGAAATTAAAGAAGCTGATCCTGAAACTGTTAATAAAAGTCTGGACACCAAGGATGTTGAAGTGGAACCTGAGCAGGCAGTGTCTGGAACTATATATGCCAATGGTGACCATTCCGGAGAAAGCGTCGAGCGAGATGTAGTGGAAGTTGAAGTCTCTGGTCAAACATCTGCTATATCAAGGTCAATCACTGGCTCAGAGCAAGAAGGAGAAGCTAAAGATCATATAGATGAAGAAGCTAACCTTGAAGGCTCAGTTTCAGATGGAGAGACAGATGGTATGATTTTTGGAAGCTCTGAAGCTGCCAAACAGTTTATGGAGGAGCTGGAAAGGGAATCTGGTGGTGGCTCCTATGCTGGTGCTGAGGTTTCTCAGGATATTGATGGTCAGATTGTCACCGACTCAGATGAGGAGGCTGATACTGATGAAGAAGGAGATGTGAAGGAGTTGTTTGATTCAGCTGCCTTAGCTGCTCTTTTAAAAGCAGCAACAGGTGGTGATTCTGATGGTGGCAACATAACAGTCACGTCTCAAGATGGATCAAGACTGTTCTCTGTTGAACGTCCTGCTGGTCTTGGGTCATCACTCCGGTCACTGAGGCCAGCTCCCCGACCAAGCCAACC

CAATCTTTTTACTCATTCCAATCTCCAGAACAGTGGAGAATCTGAGAACAACTTGAGTGAAGAAGAGAAGAAGAAACTGGACACACTACAGCAGATCAGGGTCAAGTTTTTGAGGCTTATTCACAGGTTGGGTCTATCTTCTGATGAGCCCATAGCTGCACAAGTTTTGTACCGGATGACACTTATTGCACGAAGGCAAAACAGTCCACTTTTTAGCGTTGAGGCTGCCAAGATGAAAGCTTTCCAGCTTGAAGCAGAGGGGAAAGATGATTTGGACTTCTCTGTGAATATCCTGGTTATTGGCAAATCTGGGGTGGGTAAGAGCGCTACCATAAACTCTATCTTTGGAGAGGAAAAAACATCAATTGATGCCTTTGGACCTGCTACCACCAGTGTGAAAGAGATCAGTGGTGTTGTAGATGGTGTTAAGATTCGGGTGTTTGATACACCTGGCCTCAAGTCCTCTGCGATGGAACAGGGTTTCAATCGCAGTGTCTTGTCTTCAGTAAAGAAGTTGACTAAGAAGAATCCCCCTGATATTTACCTCTATGTCGATCGGTTGGATGCCCAAACTAGAGATCTCAATGATCTTCCTATGCTGAAGACTATCACAAGTTGTCTTGGCCCTTCAATATGGCGAAGCGCCATAGTCACCCTCACACATGGAGCTTCTGCACCTCCAGATGGACCTTCTGGATCCCCTTTAAGTTATGAGGTGTT

TGTTACTCAAAGATCTCATGTTGTTCAGCAGTCCATCGGGCAAGCAGTAGGCGATTTACGAATGATGAGTCCAAGTCTGATGAATCCCGTCTCTCTGGTAGAAAATCATCCATCTTGCAGGAGGAATAGGGATGGACATAAGATACTACCTAATGGCCAGAGCTGGAGGCCTCAATTACTACTATTAAGCTACTCAATGAAGATCTTATCTGAAGCAAGTGCACTTTCAAAGCCTGAAGATCCATTTGATCACCGTAAGCTCTTTGGTTTCCGCACACGCTCACCACCTCTTCCCTACATGCTTTCTTCAATGTTGCAGTCACGTGCGCATCCAAAGCTTTCTGCTGAGCAGGGTGGTGACAACGGTGATTCAGACATTGACTTAGATGATTTGTCAGACTCTGACCAAGAAGAAGAAGATGAGTATGACCAGCTTCCTCCCTTCAAGCCTCTTCGGAAGGCTCAGCTTGCTAAGCTCAGCAAAGAACAGAGGAAGGCGTACTTTGAGGAGTATGACTACAGGGTCAAGCTCCTTCAGAAGAAACAGTTGAGAGAAGATTTAAAAAGAATGAAAGAGATGAAAAGTAAGGGAAAAGAGGCTGCAATTGACAATGGTTATGCAGAGGAAGAAGCTGATGCAGGTGCAGCAGCTCCCGTAGCAGTTCCCCTTCCTGACATGGCCCTTCCACCTTCTTTTGATAGTGATAATCCCGCCTATAGGTACCGCTTCTTGGAGCCCACATCACAGTTCCTTGCAAGGCCTGTTCTGGACACGCATGGTTGGGATCATGATTGTGGCTATGATGGTGTTAACGTGGAACAAAGTTTAGCCATTGCCAGTCGTTTCCCTGCTGCAGTTACTGTGCAAATCACCAAAGATAAGAAGGATTTCAGTATCAATTTGGACTCTTCGATTGCTGCTAAGCACGGAGAAAATGGATCAACCATGGCTGGCTTTGATATTCAAAGCATAGGGAAGCAACTTGCCTATATTGTCCGAGGAGAAACCAAATTCAAAAGCTTGAAGAAGAACAAGACTGCTTGCGGAATTTCTGTTACATTTCTAGGTGAAAATATGGTCACTGGACTTAAAGTTGAAGATCAAATCATCTTAGGCAAGCAATACGTTCTAGTTGGCAGTGCTGGCACTGTTCGATCTCAGAGTGACACAGCTTATGGGGCTAACTTTGAACTGCAGAGGAGGGAGGCAGATTTCCCAATCGGTCAGGTGCAATCTACATTGTCTATGTCCGTCATAAAGTGGAGAGGTGATTTGGCTCTAGGTTTCAACAGTATGGCGCAATTCGCTGTGGGACGCAATTCGAAGGTAGCTGTTCGAGCAGGAATCAATAACAAGCTCAGTGGGCAAGTAACCGTGAGGACAAGCAGTTCAGACCATCTCTCTCTTGCACTTACTGCTATTATTCCAACTGCAATTGGCATCTACAGGAAGCTTTGGCCGGATGCTGGCGAGAAGTACTCAATCTACTAA

>slToc159-2 (Solyc01g080780.2.1)

ATGAAACTTATCATAGATCAGTCGGACCAACAAATAGCCAATGCCGACTACGATGGAGAGGTTTCTGAGGGTCATTTACCAAAGGTTGATGCTGAGATTGTGACAGACTTAGCTGAAGAGGTAGATACAGACGAAGAGAGTGAAGAGAATGAGATGTTTGATGCTGAAGCACTGGCTATGCTGTTGAGGGCTGCTACCGGTGTTGGGCCTGAAGGTAGAAGTGTTTCAATTCCATCAGCTGATGGTACACAGGTTTCTTCTCTGGAGCTTCCTGATACCCCGGGATCCTCATTTCACTCGTCCAGACCTGGTCAGCCAACAAATGCAGATAAGTTTCCCCTATCTGATAATAAGACTGAAGGTATATCAGAAGTGATTTTGTCTGAAGAAGAAAAGAAGAAGCTTGAGAAATTACAGCAGTTAAGAATAACATTTTTGCGGCTCGTCCACAAGCTAAACCGGTCTCCTGAAGATTCCATAGCTGCACAGGTCTTATACCGGTTGGTTCGTGCTGCAGGGAAGTCAGCCTCTCAAGTATTGAGCCTTGACTCTGACCAGAAGGTAGCTATAGAGTTGGAAGCAGAGGATACAGACAGTTTGAATTTTTCTCTGAATATCCTGGTTATTGGTAAAACAGGAGTTGGTAAAAGTGCAACAATAAACTCTATCTTTGGTGAAGCCAAATCAATGGTAGATGCATTTGTACCTGCGACTACCGAT

GTGAAGGAGATAATTGGACAACTGGATGGAGTTACATTGAACATCTTGGATACTCCTGGTTTCAGATCTTCCCTGACAGAACAATCCATTAACCGAAGAACTTTGTTGTCCATAAAGAAATATATGAAGAAATACAGTCCTGATGTAGTCCTCTATGTCGACCGCATTGACACACAGTCTAGAGATCTTGGTGATTTACCATTATTCAAGTCCATCAGTAGTTATCTTGGTCCATCAATATGGCGTAATGCCATTGTTACCCTGACACATGCTGCTTCAAGTCCTCCAGATGGACCCTCGGGGCATCCTGTAAGCTATGAAATGTTTGTTGCTCAGTGCTCCCGTATCATTCAACAGTTAATTGATCACTCCATAGGTGATCCACACACGATGAATGCTGGGTTGATGAGTCTTCCATTTGCTCTTGTTGAAAACCACCCAGTCAGTCCAAAGAATGACAAAGGAGATATATTGCTTCCAAATGGAGAAAATTGGAGATCGCAGCTTCTGCTTCTGTGTTACTCAATAAAGATTTTATCAGAAGTAGATTCCATCATGAAAGATCAAGATCTTCATGACCACAGAAAGCTTTTTGGCTTCCCCAAGCGTTCACTCCCTTTACCATACTTTTTATCTTCACTGTTACAATCAAATGTTCATCCTAAAGTCTCTAACAATCAAGTTGGTGGGGATATAGGCTCAGACATTGAGCTGGTACATTCATCTGATTCCGATCAAGAAGTTGATGATTACGATGACCTTCCACCTTTCAGACCTTTGAGGAAATCTCAAATTGCTAAGCTGAGCAAGGAGCAGAAGAGAGCATATTTTGACGAGTATGATTACCGTGTGAAGCTGTTTCAGAAGAAACAATGGAGGGAGGAGTTAAAAAGGCTTCGGGACATGAAGAAGAAAGGCAAGGCAGAGATAGGTGATTACATGGAAGAAGGTGCTGACCAGGAAACAGGGAGCCAAGCAGGAGCAGCAATCCCTTTACCCGACATGGTGCTCCCAAATTCTTTTGACGGGGACAACCCAACTTACAGATACCGGTATCTAGAACCTTCGTCTCAACTTCTTGCAAGGCCTGTTATGGACTCCCAGAGCTGGGACCATGATTGTGGATATGATGGTGTGAGCATTGAGGATCACCTTGCCATTGCTGGCCAGTTTCCTGCAGTAATAGTTCTTCAGCTCACAAAGGACAAGAAAGAGTTCAACATCCACTTGGATTCATCTGTTTCAGCAAAGACGGGGAAGAAAGGATCAAGTATGGTAGGGTTCGACATTCAAACTGTAGGAAAGCAACTTGCCTACATTTTGAAGGGAGAAACAAAAGTGAAAAATCTGAAAACGAATAAAACAGCTGCAGGGATATCCATTACTTTCTTGGGTGACACTTTAGTGACGGGATTGAAATTGGAGGACCAATTTTCCATTGGTAAACAGTTAGTTGTGGTGGGAAGTACTGGCACCATCATGTCTCAGGGTAATGCAGCATATGGAGCCAACTTAGAGTTGCGTCTGAGAGAGAAAGATTACCCTGTTGGACAGGACCAAAGTTCACTCGGCCTTTCTTTGATGAAATGGAGAAACGATCTCATATGGGGATGCAATCTGCAATCTCAGTTCTCTGTTGGAAGGAATTCCAAGATTGCTGTTAGGGCCGGATTAAACAGCAAGAAAAGCGGGCAAATCACAGTTAGGACAAGCACCTCAGATCAACTATTGATTGCTATTGTAGGACTTCTGCCAATTGCTAGAGCAATAATGATGACCCTTTTTCCTCAGACAAGTGGAAAGAACTTAATATAG

>slToc159-3 (Solyc11g043010.1.1)

ATGGAGCTTGTAAATTTGAAGTACAAGGTTCAGGAGTACACCGAGGAGCGGAAGGATGTTCTGAACTGTGATGCTACTGATCTTCAATATTTGAAAGAAGAGTCACCTGATGTGGCTTTACTGAAGTCAAATGACAAGGTTCGGGACTATGCGGAGGAGCAGAAGGATACTATTGATCATCAAGTATTAACGAGAAAAGACTTACCTGCTGTAAGACTAGGGAATTTGATTGATGAGGTTCAGCTATTCATGGAGCAGCAAAAGGATGATCTGACTTGTGATGCTACTGATGATCAAATCCTCAATGATAAAGAGTCACCTGCTGTGGGGTTAGCGATTTCAAATGACAAGGTTGATGATCAGAAAGATGCTTCAGTATTTTGCACTTCAGCTTCTGAAAACCGCACTGTAAATTTATCAAGAGAGACTGTGACTGCAGAGGATGAGAATTGGAAATCATCGGAAGGTTCAGTATCCAAGAACAATGGAAAGGTTGGTGTAAACACAGCTGAGTTTGTTTCAAACCAAAGCAAAATGTTTACTGGGGTGAGAGAAACAGAAGCTGGATCTGCAAGAAATCTCGTATCATCCTCTGGTTCATCCATGACTCGTATACCACCACCTGCTCAACCGGTTGGCCTTGGTCGTGCTGCCCCACTATTGGAACCTTCTCCCCAAGTGGTTCAGCAGGCTTGGGTGCATGAAGCCGCGTCTTCAGTA

CAAAACCAACTTGTTGAAGAACCCACAAATGGAGAATCTGAGGAATATGATGAGATACGCGAGAAACTCCAGATGATAAGAGTTAAGTTTTTGCGTCTTGCTCATAAGGTTGGGCAGAATCCACATAATGTAGTTGTTGCCCAGGTCCTTTATAGATTAGGATTGGATGAACAGATGAGGGGGAGAAATGGAAGTCGTGTTGCTGCTTTTAGCTTTGATCGTCCAAGTGCCATGGCCGAGCAACTTGAGGCAGCTGGACAAGAAGCCTTGGATTTCTCCTGCACAATTATGGTTCTTGGAAAAACAGGTGTTGGTAAAAGTGCGACCATAAATTCTATATTTGGTGAAGCTAAGTTTGGTAGTGATGCATTTCAGATAGGGAAAAAGAAGGTTCAAGATGTTGTTGGGACTGTGCAGGGAATTAAGGTTCGGGAAACATCATCAGATATTGTACTGTATCTCGACAGGTTGGATATGCAAAGTAGAGATTATGGTGACTTGACATTGTTGCGGACAATTACTGAAGTTCTTGGCTCATCAATATGGTTCAATACAATAGTTGTTCTTACTCAAGCTGCATCTGCTCCTCCTGAAATCTCCAATGCAATATGGCAGGCAGCAGGAGATGTGTGCCTTATGAATCCTGTTTTTCTGGTCGAGAATCACTCAAAATGCAGAACAAACAGGGTTGAGCAGAGAGTTTTACCTAATGGTCAAGTTTGGAAGCCTCACTTGTTGCTTCTGTCATTTGCATCCAAAATTTTGGCTGAGACAAGCACATTGCTGGAGTTACGAGACAGTCCACCAGATGAATCAGAATATGATCAACTGCCAGCTTTCAAACCTTTGACTAAAGTCCAGCTAGCAAAGTTGAGCCAAGAGCAAAAGAAGACATACAATGATGAATTAGAATACAGGGAAAAGCTTTTTATGAAAAAGCAATTGAAGGAGGAGAGAAAGAGACGGAGAATGATGAAGAAAATGCATGCTGCAACTAAGGATTTGCCTATGGATACCAACGAAACAGTAGAAGAGGAGACTGGCAGTGCAGCGTCTGTTCGAGTACCAATGCTAGATTTTGCCTTACCTGCTTCTTTTGATTCTGATAACCCAACTCATAGATACCGTTATCTTGATTCTTCAAATCAGTGTCTTGTGAGGCCTGTTCTAGAGCCTAATGGTTGGGACGATAATGTTGGTTATGAGGGCATGAATGTAGAAAGGTTGTTCGTTATCAAAGACAAGATACCTTTGTCTTTCTCCAGTCAGCTTTCTACGGACAAAAAGGACGGCAATCTTCAAATGGAAATATCGGTTGGTAAAGACCTTGCTTACACTTTACGCAGTGAGACTAGATTTAGCAATTACAGGAAAAATAAGGCAACAGCTGGTCTCTCAGTAACTCTCTTAGGTGATGTTATGACAAGTGGGGTGAAAGTTGAGGACAAATTGATCGTTAACAAATGA
