# Supplementary material for: Molecular Characterization and Expression Analysis of Chloroplast Protein Import Components in Tomato (Solanum lycopersicum)
Source: PLoS One. 2014 Apr 21;9(4):e95088. doi: 10.1371/journal.pone.0095088 (PMC3994019; doi:10.1371/journal.pone.0095088)
Supplement: Data S3 — The cDNA sequences of each NCBI identified tomato Toc GTPase. (DOCX) [file pone.0095088.s008.docx]

>slToc34-like-1 (XM_004235160.1)

ATGGCATCTCAAATAATTAGAGAATGGGCTGGAATACAGCAGTTTCCAGCTGCCACTCAATCAAAGTTGCTTGAATTATTGGGGAAACTGAAGCAGGAGAATGTCAGTACTCTCACAATTCTAGTAATGGGGAAAGGCGGTGTTGGAAAATCATCAACGGTGAACTCAATTATTGGGGAAAGAGCAGTTGCTGTTAGTGCATTTCAGTCAGAAACTCCCAGACCGGTGATGGTTTCGCGTTCACGAGCAGGTTTTACACTAAACATAATTGACACCCCAGGGCTAGTTGAAGGAGGATACGTCAATGACCAGGCTCTTGATCTCATAAAGAAGTTCCTCTTGAACAAGACAATTGATGTTTTGCTATATGTGGATCGTCTGGATGCATATAGAGTGGATAACTTGGATAAGCAGATTGTGAAGGCTATTACTGATATTTTTGGCAAGGAAATGTGGTGCCGAGGAATTGTGGTTCTCACTCATGCTCAGCTTTCCCCTCCTGATGGATTGACTTATGAAGAATTCACGTCCCGAAGATCAGAGGCACTTTTGAAAATCGTCCGGATGGGTGCTCGAATCAGGAAACAAGACATTCAGGCTGCTTCAATTCCTGTTGTCTTGGTTGAGAATAGTGGCAGATGTAACAAGAATGAAAGTGACGAAAAGATTCTTCCAAGTGGAATTGCGTGGATACCCAATTTAGTCCAAACTATTACAGATGCTGTTTTAAGTGGAAGCAAGGGTATTTTGGTTGATCAAAAACTGATTGAAGGTCCTAATCCAAACAATAGGGGCAAAGTGCTAATTCCTTTTATACTAGCATTCCAGTATTTCTTTGTAGTGAAAAGGATCCAGAGGTCCATTAAGAATGATATTGCACGGGAGACCAGGCCTTCATGGGCATAA

>slToc34-like-2 (XM_004239929.1)

ATGGCATCTCAGGTGATAAGAGAATGGGTTGGATTTCAGCAGTTTCCTTCAGCCACTCAGTCAAAGTTAATTGAATTGATAAGAAAATTGAAGCAGGAGAGTGTAAGTACAGTGACAATCCTAGTAATGGGGAAAGGTGGTGTTGGAAAATCTTCAACTGTAAACTCAATTTTAGGGGAAAGAGCAGTTGCTGTCAGTGCGTTTCAGTCAGAAACTCCAAGACCAGTGATGGTTTCACGTTCATGGGCAGAATTTACATTGAACATTATCGACACCCCCGGGCTGGTTGAAGGAGGATATGTCAATGACCAGGCTCTTGATCTCATAAAGAGGTTCCTCCTGAACAAGACAATTGATGTTTTGCTTTATGTGGATCGTCTTGATACATATAGGGTGGACAATTTGGATAGGCAGATTGTAAAGGCCATAACAGATAGTTTCGGCAAGGAAATATGGCGTAGAGGACTTGTGGTCCTCACACATGCTCAGGTCTCCCCTCCTGATGGATTGAGTTATGACGAGTTCACTTCGAGAAGATCAGAGGCACTTCTGAAAATTGTCCGCCTGGGAGCTCGAATGAAGAAACAAGAGATCAAGGCTGCTTCAATTCCTCTTGTTTGCGTTGAGAACAGTGGGAGATGTAACAAGAATGAACTCGACGAAAAGATTCTTCCAAATGGAACTGCTTGGATACCCAGTATACTCCAAACTATTACCGAAGTTGTCGTAAGTCAAAGCAAGGGTATCTTGATTGATCAAAAATTGATCGAAGGACCAAATCCCAACAATAAGGGCAAATTGCTGATCCCTCTTATCGCAACATTCCAGTATTTCTTCGTTGTGAAAAGGATCCAGACGTGGATCAAAAACGACATTTCAAGAGAGAACAGACCTTCATGGGCATAA

>slToc159-like-1 (XM_004247489.1)

ATGGATTCTGAAGAAGCGACGTTTTCGCCTCCTGCTGTTTCTTCTTCTCCAGGTTCTTCTCCCATCAACAATTCTTCTTCTAATCATACTGAAACTGAAAATGTCTCCAAAATTAATGTAGAAATCAATGATTCCGATATTAATAGTAATAGCAATAGTGAGGGTAAAAGCGCTAGTGATGTAACTATTGTGGGTGGTCAGCAAGAATTGCCAATTCCTGCTGACCCAGATGAGGGAACCCTAGAAAAAACTATTGGGGAAGAGAAGTTGGATGATTCCGTTGTGGGTTCTGCGGAAATTGAGAAGCCTGTTTCTGAGGTTTCTATGAGTGAGGGTGTTGAAAATGTTGAGGCTTTAGGGGGAGATGTTGGTGGTTCTGTTCCTGTTATTGGGAATAGTTTACCTGATTCTACTGACTCTGATGCTACCAAATCACTGGGGACGGGAATCGAGGGTTCGGAGGGTAATACTGAAGAGTTTGACTCGGTTGATAAGTTGAATTCAATTGAGCAGGTGAAAGATAATGGTGGTGAGGTTGCGGTTGGTGCAGGATTGAAAGAGGGTGAGGATAGGTCCCTCAGGAGGAGGTGAAGGAAACTGTGGAGGATGAAAAGATGGAACCGAAAGAGGGCGGGGATAGGTCTATCGAGGAGGAGGTGAAGGAGACTGTGGAGGATGAAAAGATAGAATTGCAAGGGGGTGAGGATAGGTCCATTCAGGAGGAGGTGAAGGAGATTGTGGAGGATGAAAAGAATGAAGCTTTAACTAGTGTTGCTTCATCTAATTTAAAGGAGGCTGAGGAACCTACCTCGGTCATCGAAGAGAGTGCTATTGCTTCATCCAATTTGAAAGAGTCTGAGGAACCTACCTCGGTCTTTGAAGAGGTTGCTATTGCTTCATCCAATTTGAAGGAGGCCGAAGAACCTACCTCGGTCATTGAAGAGAGAGCTATACATAGTGATGATGCCGAAAAACTCAATAAGGTGGTTGTTGAACAACCGTCGGAATCTTTGTTGGCTGAAACAGGTAGTAAGAAATTTACTTCCGAAGGAGATGCAGTTGTGGATGCTATTGAAGTCAATGTCTCAGGGCCAGGGGTTGCTGTTGTTGGAGATGTGGACGAGAGCAAGGAAGTGGAAGAACATATTGAAGGTACCCATGATGAAAATGTGACATCAGTAAATGATGTTGGTGAGACCAGACAACTTATTGAAGAAGTGGCTAAAATGACAGTTGATGAAGTAGATGCACAGAACCCTAAGCCTGTGGTGGATGATACTGTTGCAACTGCAGAATCAAAGCCTGTGGATAACATTGTTGGTGCTGGAAAACTTGATTCTGGAGTTGTTCAGACTGGTGATGTGGTAGCTGTTACTGAGGAAATTAAAGAAGCTGATCCTGAAACTGTTAATAAAAGTCTGGACACCAAGGATGTTGAAGTGGAACCTGAGCAGGCAGTGTCTGGAACTATATATGCCAATGGTGACCATTCCGGAGAAAGCGTCGAGCGAGATGTAGTGGAAGTTGAAGTCTCTGGTCAAACATCTGCTATATCAAGGTCAATCACTGGCTCAGAGCAAGAAGGAGAAGCTAAAGATCATATAGATGAAGAAGCTAACCTTGAAGGCTCAGTTTCAGATGGAGAGACAGATGGTATGATTTTTGGAAGCTCTGAAGCTGCCAAACAGTTTATGGAGGAGCTGGAAAGGGAATCTGGTGGTGGCTCCTATGCTGGTGCTGAGGTTTCTCAGGATATTGATGGTCAGATTGTCACCGACTCAGATGAGGAGGCTGATACTGATGAAGAAGGAGATGTGAAGGATCCACTTTTTAGCGTTGAGGCTGCCAAGATGAAAGCTTTCCAGCTTGAAGCAGAGGGGAAAGATGATTTGGACTTCTCTGTGAATATCCTGGTTATTGGCAAATCTGGGGTGGGTAAGAGCGCTACCATAAACTCTATCTTTGGAGAGGAAAAAACATCAATTGATGCCTTTGGACCTGCTACCACCAGTGTGAAAGAGATCAGTGGTGTTGTAGATGGTGTTAAGATTCGGGTGTTTGATACACCTGGCCTCAAGTCCTCTGCGATGGAACAGGGTTTCAATCGCAGTGTCTTGTCTTCAGTAAAGAAGTTGACTAAGAAGAATCCCCCTGATATTTACCTCTATGTCGATCGGTTGGATGCCCAAACTAGAGATCTCAATGATCTTCCTATGCTGAAGACTATCACAAGTTGTCTTGGCCCTTCAATATGGCGAAGCGCCATAGTCACCCTCACACATGGAGCTTCTGCACCTCCAGATGGACCTTCTGGATCCCCTTTAAGTTATGAGGTGTTTGTTACTCAAAGATCTCATGTTGTTCAGCAGTCCATCGGGCAAGCAGTAGGCGATTTACGAATGATGAGTCCAAGTCTGATGAATCCCGTCTCTCTGGTAGAAAATCATCCATCTTGCAGGAGGAATAGGGATGGACATAAGATACTACCTAATGGCCAGAGCTGGAGGCCTCAATTACTACTATTAAGCTACTCAATGAAGATCTTATCTGAAGCAAGTGCACTTTCAAAGCCTGAAGATCCATTTGATCACCGTAAGCTCTTTGGTTTCCGCACACGCTCACCACCTCTTCCCTACATGCTTTCTTCAATGTTGCAGTCACGTGCGCATCCAAAGCTTTCTGCTGAGCAGGGTGGTGACAACGGTGATTCAGACATTGACTTAGATGATTTGTCAGACTCTGACCAAGAAGAAGAAGATGAGTATGACCAGCTTCCTCCCTTCAAGCCTCTTCGGAAGG

CTCAGCTTGCTAAGCTCAGCAAAGAACAGAGGAAGGCGTACTTTGAGGAGTATGACTACAGGGTCAAGCT

CCTTCAGAAGAAACAGTTGAGAGAAGATTTAAAAAGAATGAAAGAGATGAAAAGTAAGGGAAAAGAGGCTGCAATTGACAATGGTTATGCAGAGGAAGAAGCTGATGCAGGTGCAGCAGCTCCCGTAGCAGTTCCCCTTC

CTGACATGGCCCTTCCACCTTCTTTTGATAGTGATAATCCCGCCTATAGGTACCGCTTCTTGGAGCCCACATCACAGTTCCTTGCAAGGCCTGTTCTGGACACGCATGGTTGGGATCATGATTGTGGCTATGATGGTGTTAACGTGGAACAAAGTTTAGCCATTGCCAGTCGTTTCCCTGCTGCAGTTACTGTGCAAATCACCAAAGATAAGAAGGATTTCAGTATCAATTTGGACTCTTCGATTGCTGCTAAGCACGGAGAAAATGGATCAACCATGGCTGGCTTTGATATTCAAAGCATAGGGAAGCAACTTGCCTATATTGTCCGAGGAGAAACCAAATTCAAAAGCTTGAAGAAGAACAAGACTGCTTGCGGAATTTCTGTTACATTTCTAGGTGAAAATATGGTCACTGGACTTAAAGTTGAAGATCAAATCATCTTAGGCAAGCAATACGTTCTAGTTGGCAGTGCTGGCACTGTTCGATCTCAGAGTGACACAGCTTATGGGGCTAACTTTGAACTGCAGAGGAGGGAGGCAGATTTCCCAATCGGTCAGGTGCAATCTACATTGTCTATGTCCGTCATAAAGTGGAGAGGTGATTTGGCTCTAGGTTTCAACAGTATGGCGCAATTCGCTGTGGGACGCAATTCGAAGGTAGCTGTTCGAGCAGGAATCAATAACAAGCTCAGTGGGCAAGTAACCGTGAGGACAAGCAGTTCAGACCATCTCTCTCTTGCACTTACTGCTATTATTCCAACTGCAATTGGCATCTACAGGAAGCTTTGGCCGGATGCTGGCGAGAAGTACTCAATCTACTAA

>slToc159-like-2 (XM_004229523.1)

ATGGACTCAAAGAATTATGGTGTTCCACTTTCTACAACACAAGGGAGCCCACCTGCAAATGTCCCTCTTTTCACTTCAACAGGTATTAGAGCTCCTATTACTATTGATGATTCCGACTTTGAATACTCTGTCTCTGTTAATGGACCAAAAAATAAGTCTGAGAATGCGATACAATCTGGGATGGAAGAGGACTTGATCATTACAAAAACTGTTGAGATGGTTTTGGATTCCGACAGTATTGAAGATGATAAAGCTGAAATTGACACTGTTCAGAATTTCATAACGGAGGGAGGTGAGGCCAATTATACTTTAGAAGGAGGAAGAGATTTGTATCAGGTTGCAAGTAAAGAATCTACTTTGTTGGGACAAGAGTTGGACAATGATAAAATGCTTTTAGCAGAAGAAAGTCAAATTCTTGATAGTAGTGTAGAGTCAAAAAGTGCAGATTCGTCTCGTGGTGTTAAGCCTTCTGATACTGCTTACTCCAGCCCAAGGGAAGAAAATAGAGTATCAAAATTTGGAGCCGATGATTTGAACTCTGGAAACAGCATCATCTTACATACTGGAGCTTCCGGGGACAGTCAGAAATCAGAAAGTAAGGAGGATGGGGTTTATCAAGGAAGTGATTGTCAGGATATTGCCACTAGAACCGAAACCGAATCTTATCATGAACCCATCAAGGATAGTGAAGCTGAGAGCTTGGAGTGCATTGATATTTCAGTACCATCAACAGCTGAAGAACAAGTATATTCTTCATCAGATGTAACTTGGTCCACCAGAGCTGAAGATGATCTACCTAAGTTATCGGATAAAACACAACACAGGGAGGCTCGTTTAAATCCAGACTTAGAAGCTAAATGCAAAGACATTGATACAGTCAAATTGTTCAAAAATGAAGAAGCCCTATTCTTACATGAAAATGATGAAAGTTTGACCTTTGATGGATCCGGTGGTATGAAACTTATCATAGATCAGTCGGACCAACAAATAGCCAATGCCGACTACGATGGAGAGGTTTCTGAGGGTCATTTACCAAAGGTTGATGCTGAGATTGTGACAGACTTAGCTGAAGAGGTAGATACAGACGAAGAGAGTGAAGAGAATGAGATGTTTGATGCTGAAGCACTGGCTATGCTGTTGAGGGCTGCTACCGGTGTTGGGCCTGAAGGTAGAAGTGTTTCAATTCCATCAGCTGATGGTACACAGGTTTCTTCTCTGGAGCTTCCTGATACCCCGGGATCCTCATTTCACTCGTCCAGACCTGGTCAGCCAACAAATGCAGATAAGTTTCCCCTATCTGATAATAAGACTGAAGGTATATCAGAAGTGATTTTGTCTGAAGAAGAAAAGAAGAAGCTTGAGAAATTACAGCAGTTAAGAATAACATTTTTGCGGCTCGTCCACAAGCTAAACCGGTCTCCTGAAGATTCCATAGCTGCACAGGTCTTATACCGGTTGGTTCGTGCTGCAGGGAAGTCAGCCTCTCAAGTATTGAGCCTTGACTCTGACCAGAAGGTAGCTATAGAGTTGGAAGCAGAGGATACAGACAGTTTGAATTTTTCTCTGAATATCCTGGTTATTGGTAAAACAGGAGTTGGTAAAAGTGCAACAATAAACTCTATCTTTGGTGAAGCCAAATCAATGGTAGATGCATTTGTACCTGCGACTACCGATGTGAAGGAGATAATTGGACAACTGGATGGAGTTACATTGAACATCTTGGATACTCCTGGTTTCAGATCTTCCCTGACAGAACAATCCATTAACCGAAGAACTTTGTTGTCCATAAAGAAATATATGAAGAAATACAGTCCTGATGTAGTCCTCTATGTCGACCGCATTGACACACAGTCTAGAGATCTTGGTGATTTACCATTATTCAAGTCCATCAGTAGTTATCTTGGTCCATCAATATGGCGTAATGCCATTGTTACCCTGACACATGCTGCTTCAAGTCCTCCAGATGGACCCTCGGGGCATCCTGTAAGCTATGAAATGTTTGTTGCTCAGTGCTCCCGTATCATTCAACAGTTAATTGATCACTCCATAGGTGATCCACACACGATGAATGCTGGGTTGATGAGTCTTCCATTTGCTCTTGTTGAAAACCACCCAGTCAGTCCAAAGAATGACAAAGGAGATATATTGCTTCCAAATGGAGAAAATTGGAGATCGCAGCTTCTGCTTCTGTGTTACTCAATAAAGATTTTATCAGAAGTAGATTCCATCATGAAAGATCAAGATCTTCATGACCACAGAAAGCTTTTTGGCTTCCCCAAGCGTTCACTCCCTTTACCATACTTTTTATCTTCACTGTTACAATCAAATGTTCATCCTAAAGTCTCTAACAATCAAGTTGGTGGGGATATAGGCTCAGACATTGAGCTGGTACATTCATCTGATTCCGATCAAGAAGTTGATGATTACGATGACCTTCCACCTTTCAGACCTTTGAGGAAATCTCAAATTGCTAAGCTGAGCAAGGAGCAGAAGAGAGCATATTTTGACGAGTATGATTACCGTGTGAAGCTGTTTCAGAAGAAACAATGGAGGGAGGAGTTAAAAAGGCTTCGGGACATGAAGAAGAAAGGCAAGGCAGAGATAGGTGATTACATGGAAGAAGGTGCTGACCAGGAAACAGGGAGCCAAGCAGGAGCAGCAATCCCTTTACCCGACATGGTGCTCCCAAATTCTTTTGACGGGGACAACCCAACTTACAGATACCGGTATCTAGAACCTTCGTCTCAACTTCTTGCAAGGCCTGTTATGGACTCCCAGAGCTGGGACCATGATTGTGGATATGATGGTGTGAGCATTGAGGATCACCTTGCCATTGCTGGCCAGTTTCCTGCAGTAATAGTTCTTCAGCTCACAAAGGACAAGAAAGAGTTCAACATCCACTTGGATTCATCTGTTTCAGCAAAGACGGGGAAGAAAGGATCAAGTATGGTAGGGTTCGACATTCAAACTGTAGGAAAGCAACTTGCCTACATTTTGAAGGGAGAAACAAAAGTGAAAAATCTGAAAACGAATAAAACAGCTGCAGGGATATCCATTACTTTCTTGGGTGACACTTTAGTGACGGGATTGAAATTGGAGGACCAATTTTCCATTGGTAAACAGTTAGTTGTGGTGGGAAGTACTGGCACCATCATGTCTCAGGGTAATGCAGCATATGGAGCCAACTTAGAGTTGCGTCTGAGAGAGAAAGATTACCCTGTTGGACAGGACCAAAGTTCACTCGGCCTTTCTTTGATGAAATGGAGAAACGATCTCATATGGGGATGCAATCTGCAATCTCAGTTCTCTGTTGGAAGGAATTCCAAGATTGCTGTTAGGGCCGGATTAAACAGCAAGAAAAGCGGGCAAATCACAGTTAGGACAAGCACCTCAGATCAACTATTGATTGCTATTGTAGGACTTCTGCCAATTGCTAGAGCAATAATGATGACCCTTTTTCCTCAGACAAGTGGAAAGAACTTAATATAG

>slToc159-like-3 (XM_004230964.1)

ATGATGGAAAATGGGGAGGAAAGATTTGGCAAGGCCCGAATGGACGACTGGAATGGTGTAGTAGATGAAA

CTGTTGAAGTGCGCCCGGAGGATAAGGTAGTTGTGGTGTCACATGTATCAAAGGAATCGGAAGGGGATGAAGTATTTGAGGAGGCAATAGAGCCAGAATCTCCTGGTTTTGCTGTTGAGGATGTAGTTGCTAGTGAGGGAAGAAATGATGATAACTCTGGGGATATTAATAGTTCTATTGAGGACAGTAGCAACTCTGAGTCAAGGGACAATGTTGAGAACTTTGAGGAAGCAGTTGAAGTTTTACATGAGATACAGCATGCCAATGATGAGTCTAATCAGAAGACAGATGTTATCTTGAAAGAGGAGCCTTCAGTTGAGAAGGAAAGTTGTCATGAAATCGCTGCACCGGATGAAACAGAAGTGGTTGAGAAGAATATCAAAGTAGGGAAGGGCAAAGATGACATGTCTGAGGTTGCAGATCTGGGTGCTGCTATAGAAACTGAAACTTCAGTGAATTGGGATGAAAGGAAAGATAATTCTGGCGAACCAACTGAATTTGAGAATGGAGTTTTCAATCATGTGAACTTGGGAGAAACTCAAAGTGATGATGCAAAGAAAACAATTTCTGATCAACAGGATGCCGATGAAGCGAAAGCGGGTAATAATGTTCTGCAGAACCAGGTTCATTCATACAAAGATGCATTGCTGCATGATGAAGATAATGTTGATGTCATTGAAACATCTGCTGTTCAGCCTGCTGGACATCAGGACACTGCTGACGTCCACAACAATGTCTCAGATAGTTCTGGCTCTGTGCTGAAAGATGAAGGAGATACCGAATGGGAAGGAGTGCTTAAATCTCTGGATTCTGATGTTAAGGATGAAGAGCAGAAAGATATTTCTCCTAATGATGCTTCCACAAATGGTCACCACAGTGAAAGCTTGAACCCAAGTGATGAACTCAAAGAAGAAGCAGGGCCTAGTCCAGAAAGGATTAACGGTTATAATATGAATGAAGAGCAGAGAGATGTAGAAAGAACAGTGCCTAGTCCAGAACTAGTTAACGGTAGTAACAAGGATGAGGAGCAGCAGATAGATGGAGTAAAAGCAGTGCATAGTCCAGAACCCGTTAATGGAAGTAATAAGGATGAAGAGCAGCAGATAGATGGAGTAAAAGCCATTAGTCCAGAACCAGTCAACGGTAGTAATAAAGTTGAAGGGCAGCAGTTAGATGGAGAAAAGGCCGTGTGTAGTCCAGAACCAATTAACTGTACTAATAAGGATGAACAGCAGATAGATGATCAAGACAACGACTCTGTTTCTATTCTACAAGGAGGTCACTTCCCCTTAAAGGCAGAAGTGACAGAAAAAGAATCAACTGGTCCAGAACTAATGGGTGATGCTTCTGATCATCAAGGACTGAAATTAAATGAGTCCCCTACTATGGAACCAGGAAATTTGAATGACAGGACCAATGAGCAGAAAGATGTTTCGGTTTCTGACTCTTCAGCTTCTTTGAACCATTCCGGAATTTCAGTTAGGGGTAAAGTGACTGCAGATGATGAAATGTCTAAATCATCAGAAGCATTGCCCTCTGACAATAATGAAAAGGTCTCGAAGGTTTCTCAAGATGCAGTTGTTGGAGTTGATAAAGTTGTAGAGAAGGAATCGGTTGATAAAGTCATAGAGAAGGAACCGGTGTCAGTTGTTGTAAAGGACCTGAAGCAAAGTGTTCCTAGGGTTAGGGAATCTGAAGCCAGATCTGCAACGGAGCATCCATCATCTTCCAATGCATCTGCAACTCGGATACCGGCACCTGCCGGCCTTGGTCGTGCTGCCCCACTATTGGAACCTGCTCCCCGAGTGGTTCAGCAGCCTCGTGTAAATGGAACAGCATCTCCAGTACAAAACCAGCTTGTTGAAGAATCTACAAATGGAGAGGCTGATGAATATGATGAGACACGTGAGAAGCTCCAGATGATAAGAGTCAAGTTTTTGCGTCTAGCTCATCGGAATGGACAGACTCCACATAATGTGGTTGTTGCCCAAGTCTTGTATAGATTGGGATTGGCTGAACAGCTACGGGGGAGAAGTGGAGGTCGTGTTGGTGCTTTTAGTTTTGATCGTGCAAGTGCTATGGCTGAGCAACTCGAGGCAGCTGGACAAGAACCCCTGGACTTCTCTTGCACAATAATGGTTCTTGGAAAAACTGGTGTTGGTAAAAGTGCAACCATAAATTCGATATTTGATGAAGTTAAGTTTGACACTGATGCTTTCCAGGTAGGGACAAAAAAGGTTCAGGATGTTGTTGGCACTGTGCAGGGAATCAAGGTTCGGGTGATTGACACTCCTGGGCTTCTTCCTTCCTGGTCAGACCAGCGCCAGAATGAGAAGATCCTTCATTCTGTCAAACGGTTTATTAAGAAGACACCCCCAGATATTGTTCTGTATCTTGACAGGTTGGATATGCAGAGCAGAGATAATGGTGATATGCCATTGTTGAGGACAATTACTGAAATTTTTGGACCATCAATATGGTTTAATGCCATAGTTGTTCTGACACATGCTGCGTCTGCTCCACCTGAAGGCCCCAATGGTACTGTAACAAGCTATGACATGTTTGTAACTCAGCGCTCTCATGTTGTCCAGCAAGCTATACGTCAGGCTGCTGGAGATATGCGCCTTATGAATCCTGTTTCGCTAGTTGAGAATCACTCAGCATGCAGGACTAACAGGGCTGGACAGAGGGTGCTGCCTAATGGACAGGTTTGGAGGCCTCACTTGTTACTTCTGTCATTTGCATCAAAAATTTTGGCTGAAGCAAACACATTGCTTAAATTACAAGATAGTAGCGCACCAGGACAGCCTTATGCCACTAGAACAAGATCCCCTCCTTTACCTTTCCTATTGTCATCCCTTCTACAGTCA

AGGCCACAGGTTAAACTACCAGCAGAGCAATTTGATGATGATGATGACGCTTTAGATGATGATCTGGATGAGTCTTCAGAGTCAGAAGATGAATCAGAATATGATCAACTGCCACCTTTCAAGCGCTTGACCAAAGCACAGCTGGCAAAGTTGTCCAAAGAGCAGAAGAAGGCCTACAATGATGAGTTAGAATACAGGGAAAAGCTTTTTATGAAAAAGCAGTTGAAGGAAGAGAGGAAGAGGCGGAAGATGATGAAGAAAATGCAAGCTGCGGCTGAAAGTTTACCACCAACTGATCCCAGCGAAAATGTAGATGAAGAGACTGGTGGTGCATCATCAGTGCCAGTACCCATGCCAGACTTGGCGTTACCTGCTTCATTTGATTCTGATAATCCAACTCATAGATATCGATATCTGGATTCTTCAAATCAGTGGCTTGTGAGGCCTGTTCTAGAACCCAACGGCTGGGACCATGATGTTGGTTATGAGGGCATCAACGTAGAAAGATTGTTTGTTGTCAAAGACAAGATCCCTATATCTCTGTCCAGTCAGGTTTCAAAGGACAAAAAGGACACCAATCTTCAAATGGAAATTGCAAGTTCAGTGAAGCATGGGCATGGAAAAGCAACTTCTTTAGGTTTTGATATGCAGTCAGTTGGTAAAGACCTAGCTTATACTCTTCGCAGTGAGACCAGATTCTGCAATTATAGGAAGAATAAGGCGACAGCTGGTCTTTCAGTAACTCTCTTAGGTGACGTCATGACAGGTGGGGTGAAAATTGAGGACAGGTTAACTTTTAATAGACGGGGATCTTTGGTTGTGTCTGGGGGAGCCATGTTTGGCCGTGGTGATGCTGCTTATGGTGGTAGTTTGGAAGCGACATTGCGAGACAAAGACCATCCTCTTGGTCGTTTCCTCTCAACCTTGGGCCTCTCGGTTATGGACTGGCATGGAGATCTTGCTATCGGATGCAATTCACAAACACAGATACCTATTGGACGATACACAAACTTGATTGGCCGTGTCAACATTAACAATAAAGGATCTGGACAAGTCAGTATCCGTTTGAACAGCTCAGAACAGCTTCAGATTGCTTTGATAAGCTTAATTCCACTTGTCAGAAAGCTGATTAGCTACTCTCAACCAGCACAATATGGATGA
